# An Integrative Pharmacogenomic Approach Identifies Two-drug Combination Therapies for Personalized Cancer Medicine

**DOI:** 10.1038/srep22120

**Published:** 2016-02-26

**Authors:** Yin Liu, Teng Fei, Xiaoqi Zheng, Myles Brown, Peng Zhang, X. Shirley Liu, Haiyun Wang

**Affiliations:** 1 School of Life Science and Technology, Tongji University, Shanghai 200092, China; 2 Shanghai Key laboratory of tuberculosis, Shanghai Pulmonary Hospital, Shanghai 200433, China; 3 Department of Biostatistics and Computational Biology, Dana-Faber Cancer Institute and Harvard School of Public Health, Boston, MA 02215, USA; 4 Department of Medical Oncology, Dana-Farber Cancer Institute and Harvard Medical School, Boston, MA 02115, USA; 5 Center for Functional Cancer Epigenetics, Dana-Farber Cancer Institute, Boston, Massachusetts, MA 02115, USA; 6 Department of Mathematics, Shanghai Normal University, Shanghai 200234, China; 7 Department of Thoracic Surgery, Shanghai Pulmonary Hospital of Tongji University School of Medicine, Shanghai 200433, China

## Abstract

An individual tumor harbors multiple molecular alterations that promote cell proliferation and prevent apoptosis and differentiation. Drugs that target specific molecular alterations have been introduced into personalized cancer medicine, but their effects can be modulated by the activities of other genes or molecules. Previous studies aiming to identify multiple molecular alterations for combination therapies are limited by available data. Given the recent large scale of available pharmacogenomic data, it is possible to systematically identify multiple biomarkers that contribute jointly to drug sensitivity, and to identify combination therapies for personalized cancer medicine. In this study, we used pharmacogenomic profiling data provided from two independent cohorts in a systematic in silico investigation of perturbed genes cooperatively associated with drug sensitivity. Our study predicted many pairs of molecular biomarkers that may benefit from the use of combination therapies. One of our predicted biomarker pairs, a mutation in the BRAF gene and upregulated expression of the PIM1 gene, was experimentally validated to benefit from a therapy combining BRAF inhibitor and PIM1 inhibitor in lung cancer. This study demonstrates how pharmacogenomic data can be used to systematically identify potentially cooperative genes and provide novel insights to combination therapies in personalized cancer medicine.

Tumors are known to continuously evolve through the stepwise acquisition of molecular alterations, and individual tumors have been estimated to carry hundreds of molecular alterations^1^,^2^,^3^. Some of the acquired alterations can promote cell growth and prevent apoptosis in response to the specific tumor micro-environment. Only a subset of these molecular alterations is expected to drive the tumorigenic process and encode proteins as potential therapeutic targets. During the past decade, novel classes of drugs capable of targeting specific molecular alterations have been applied to personalized cancer medicine^4^,^5^,^6^,^7^,^8^. Today, knowledge linking a specific tumor molecular alteration (biomarker) to a particular drug has increased substantially, leading to improved efficacy in personalized medicine^9^,^10^,^11^,^12^,^13^. However, due to the complexity of genetic or epigenetic alterations associated with an individual tumor, a single alteration often fails to interpret the entire observed drug sensitivity. Very often only a subset of patients harboring the alternation will fully respond to the drug targeting it, and tumor cells sometimes become drug resistant after prolonged treatment^14^,^15^,^16^,^17^.

Many studies have identified second biomarkers that determine tumor sensitivity to anti-cancer therapies^14^,^17^,^18^,^19^,^20^,^21^,^22^,^23^. For example, while melanoma patients harboring BRAF V600E mutation respond well to BRAF inhibitors, colon cancer patients with the same mutation often don’t due to the feedback activation of EGFR and its associated signaling pathway^21^. In a reciprocal manner, EGFR inhibition is effective in most epithelial cancers with EGFR mutations, but molecular alterations of KRAS have been implicated in acquired resistance to anti-EGFR therapies in colon cancer patients^22^. In addition, EGFR T790 M secondary mutation^14^,^15^, MET amplification^17^, or expression of the MET receptor ligand HGF^23^ are also known to be involved in resistance to EGFR inhibitors in lung cancer. These studies were addressing individual hypotheses based on feedback activation associated with clinical therapies. High-throughput screening was also designed to identify potential combinations^24^. However, this study only investigated limited cancer cells, since it is impractical to screen all possible drug combinations for many cancer cells as the number of drugs increase. Based on an assumption that the combination of two drugs can improve therapeutic efficacy due to their complementary mechanism, some computational methods have been developed to predict drug combinations^25^,^26^,^27^,^28^,^29^,^30^. For example, models based on networks or pathways analysis were conducted to investigate drug combinations^28^. Compound-pair synergy was successfully predicted using computational methods based on gene expression profiles of human B cells treated with individual compounds at multiple time points and concentrations^29^,^30^. An approach taking into account various molecular and pharmacological feature of drugs predicted new drug combinations^31^. A limitation of these studies is that they relied on limited data or information between drugs and molecules. Recently two large-scale pharmacogenomic profiles, the Cancer Cell Line Encyclopedia (CCLE)^32^ and Cancer Genome Project (CGP)^33^, were reported. Both studies provided high-throughput genomic information and pharmacological profiling of anti-cancer drugs across many cancer cell lines. However, the CCLE and CGP studies focused on single agents rather than multiple genes for combination therapies. With the availability of these new data, it is now possible to systematically identify combination biomarkers that act cooperatively to determine tumor sensitivity to different targeted drugs.

In this study, we first analyzed the CCLE dataset. We applied decision tree^34^,^35^,^36^,^37^ to identify genomic alterations that contributed to drug sensitivity. We then integrated transcriptome profiles to systematically determine the cooperative influence of a given genomic alteration combined with a particular dysregulated transcript on drug sensitivity for individual cell lines. By independently integrating the results of our initial CCLE analysis with the CGP dataset, we identified a set of candidate biomarker pairs that could potentially be targeted by two drugs to improve cell sensitivity. We further validated some of our predictions either by literature or by experiments. Our approach illustrates how an integrative computational analysis integrating genomic alterations and transcription changes can identify putative combination therapies. The list of predicted candidate pairs is also a potentially useful resource for future validation by others.

## Results

### Identifying combinations of molecular alterations that modulate drug sensitivity

**Figure 1:**
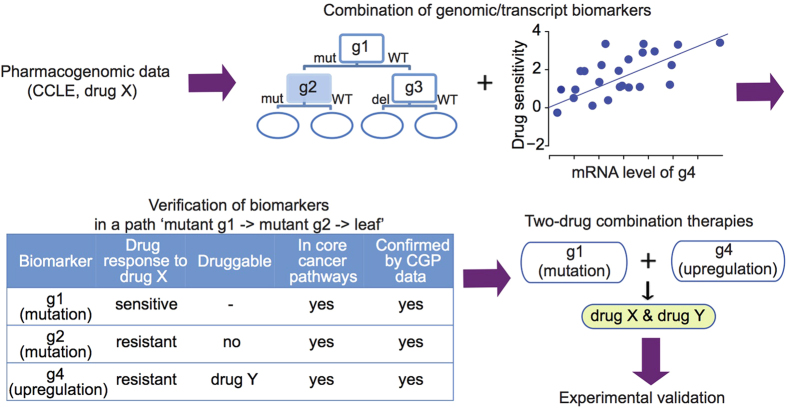
Computational approach for identifying two-drug combination therapies. Mutation status, CNV data, gene expression profiling data, and pharmacological profiles from the CCLE pharmacogenomic dataset were used to identify cooperative biomarkers associated with drug sensitivity. Genomic alterations, including mutations and CNVs, were used to generate a decision tree. Gene expression profiling data were used to identify a second gene whose altered transcript levels were associated with decreased drug sensitivity in cells with a specific genomic alteration in a first gene. To narrow down candidate biomarkers for potential drug combination therapies, gene pairs and their associated drugs identified using the CCLE dataset were independently verified using the CGP pharmacogenomics dataset, as well as information from cancer core pathways and the DrugBank database.

**Figure 2:**
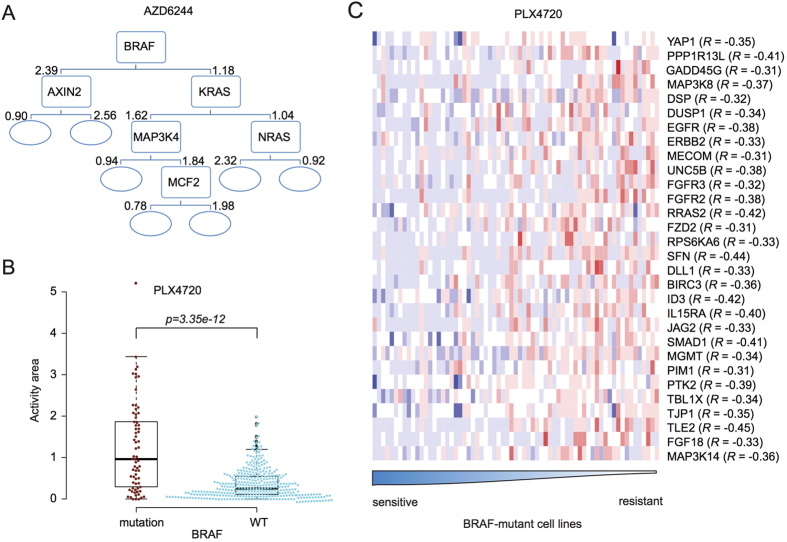
Combined effects of genomic changes and transcript alterations on drug sensitivity. (**A**) Decision tree for the MEK inhibitor AZD6244 for cancer cell lines in the CCLE dataset. The root node (top, BRAF) and intermediate nodes (AXIN2, KRAS, MAP3K4, MCF2) indicate the gene best able to classify the cells into two groups. Cells harboring the altered gene were grouped into the left branching child node, and cells harboring the wild-type gene were grouped into the right branching child node. At leaf nodes, no genes could split the cells. Values marked on the left corner of the left child node and on the right corner of the right node indicate the median drug sensitivity value for the cells in the altered and wild-type groups, respectively. A higher activity area value indicates higher drug sensitivity. (**B**) Cell lines with a BRAF mutation are more sensitive to the BRAF inhibitor PLX4720. (**C**) mRNA expression levels of 30 genes correlate with drug sensitivity in BRAF mutant cell lines. Each column in the heatmap represents a BRAF mutant cell line, which was ranked based on its sensitivity to the BRAF inhibitor PLX4720 where sensitivity decreases from left to right across each row. Each row in the heatmap represents a specific gene whose expression is normalized across the column, with high expression shown in red and low expression shown in blue.

We developed a computational approach to identify potential combination therapies that may inhibit tumor growth (Fig. 1). We started the analysis with the CCLE dataset^32^, which includes the mutation and copy number variation (CNV) status and transcriptome profiles of around 500 human cancer cell lines, and pharmacological profiles of these cell lines treated with 24 anti-cancer drugs. Drug response for each cell line is determined as the activity area, which corresponds to the area under the dose response curve such that higher values indicate stronger cell sensitivity to the drug^38^. We applied decision tree to identify genomic alterations (e.g., mutant g1, mutant g2, and deletion in g3) that may act cooperatively to influence drug sensitivity levels across the cell lines. At root nodes or intermediate nodes in the decision tree, the p value derived from the Mann-Whitney U-test for two groups of cells separated by the status of an altered gene was used as the splitting criterion. The growth of a tree was terminated when no genes could be identified that split the cells when p < 0.001 was used, or when less than five cells remained in the node. The selected genes were best able to split the cells into two groups with maximal differences in drug sensitivity between them. In the decision tree, cells harboring the altered gene were assigned to the left child node, and those with wild-type gene to the right. For example, MEK inhibitors are kinase inhibitors that decrease the ectopic activity of the ERK signaling pathway by targeting the activity of MEK kinase. In a tree for the MEK inhibitor AZD6244 (Fig. 2A), BRAF was the first gene to best split the cells. The values marked on the left and right sides of each child node correspond to the median drug sensitivity values for cells in their respective groups. BRAF mutant cells were more sensitive to AZD6244 than BRAF wild-type cells, although the sensitivity decreases in the mutant BRAF cells that also harbor AXIN2 mutation. Cells with wild-type BRAF could be further split into two groups based on KRAS mutation, as KRAS mutant cells had increased sensitivity to AZD6244. Mutations in MAP3K4 and MCF2 make the cell less sensitive to the drug in KRAS mutant cells, while mutation in NRAS make the cell more sensitive in the KRAS wild type cells. Overall, cells with mutations in BRAF, KRAS, or NRAS were sensitive to AZD6244, whereas cells with mutations in AXIN, MAP3K4, or MCF2 convey resistance to AZD6244. The same analysis on the MEK inhibitor PD-0325901 corroborated the effect of BRAF, KRAS, NRAS, and MAP3K4 in drug response (Figure. S1). These results suggest that drug sensitivity of a given cell line is the result of the combinatorial effects of multiple genomic alterations, involving genes from the root node to the leaf node in the decision tree.

We then integrated the results of our decision tree analysis with mRNA expression profiles to identify genes whose expression (e.g., upregulated g4, Fig. 1) was correlated with drug sensitivity in a specific subset of cells defined by an individual path in the decision tree (e.g., mutant g1, blue node in the decision tree in Fig. 1). We calculated Pearson correlation coefficients for comparisons between gene expression and drug sensitivity across cells at each decision tree node. We removed genes without annotations in the cancer core pathways (Table S1), since their expression may be indirectly associated with drug sensitivity. Finally, we identified a subset of genes whose expression was correlated with drug sensitivity only in cells with some particular genomic alterations. For example, cancer cells with a BRAF mutation exhibited significantly increased sensitivity to PLX4720, a BRAF inhibitor that decreases the ectopic activity of the ERK signaling pathway (Fig. 2B). Within the BRAF mutant cell lines, the mRNA expression levels of 30 genes in core cancer pathways are negatively correlated with drug sensitivity to PLX4720 (Fig. 2C, adjusted p < 0.05). We hypothesize that increased expression of these 30 genes might contribute to the limited therapeutic effects of BRAF inhibitors, such as PLX4720, in BRAF mutant cells.

Traveling down from root node to leaf node in a single path, we were able to identify a set of biomarkers that contribute jointly to drug sensitivity. In the case of the ‘mutant g1 − > mutant g2 − > leaf’ path (Fig. 1), cells carrying a mutation in g1 are sensitive to drug X, but are less sensitive if they also carry a mutation in g2 or have higher expression of g4. From the CCLE-derived genes pairs, we further focused on those where a specific drug X is effective in cells harboring a first altered gene (mutation in g1), but has limited therapeutic effects in cells harboring a second altered gene (upregulated g4). If the second gene functions in core cancer pathways and is druggable (by drug Y from DrugBank^39^, the two genes could be potentially targeted simultaneously with the X and Y combination. We further applied the same computational approach to the CGP dataset^33^ to narrow down potent genes pairs. The CGP dataset includes mutation, gene rearrangements, CNV, and expression profiles of nearly 800 cancer cell lines, and pharmacological profiling of them treated with 130 anti-cancer drugs. Drug response in this data was based on IC50 values, and lower values indicate stronger cell sensitivity to a drug. Only drug combinations were consistent between the CCLE and CGP datasets were kept for further investigation.

### Narrowing down candidate pair-wise biomarkers to identify potential two-drug combinations

Our analysis generated combination biomarkers – including genomic and transcript alterations - that act cooperatively to determine tumor sensitivity to different targeted drugs. These multiple biomarkers could guide potential drug combination therapies. Considering two drugs combination should be more relevant for clinical use, we then focused on pair-wise biomarkers that included two molecular alterations. However, still too many genes are likely to be involved in drug sensitivity. Since the genomic alterations close to the root node are genes frequently mutated in cancers, such as BRAF, which should be of better clinical potential, we then narrowed down those genomic alterations by optimally selecting the alterations associated with the first node of the tree. Furthermore, two genomic alterations for the use of combination therapies are limited by rare co-occurrence or lack of functional consequence due to genomic alterations. We further focused on pairwise biomarkers that included a frequently occurred genomic alteration as the first biomarker and a transcript alteration as the second biomarker as candidates for a two-drug combination therapy (Table S2). We annotated the candidate biomarker pairs based on several filters, including whether the gene pair was also confirmed in the independent CGP dataset, whether the two genes were both acting in the cancer core pathways, and whether the two genes are known drug targets.

**Figure 3:**
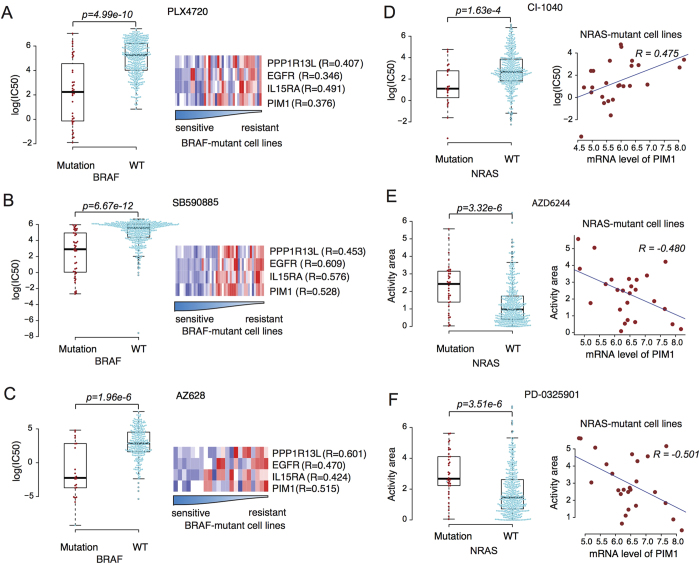
Cross-validation of cooperative biomarker pairs. Cell lines with a BRAF mutation from the independent CGP dataset were more sensitive to the BRAF inhibitors PLX4720 (**A**), left), SB590885 (**B**), left), or AZ628 (**C**), left). Higher levels of PPP1R13L, EGFR, IL15RA, or PIM1mRNA expression were associated with decreased drug sensitivity in BRAF mutant cell lines treated with the BRAF inhibitors PLX4720 (**A**), right), SB590885 (**B**), right), or AZ628 (**C**), right). NRAS mutant cell lines from the CGP dataset were more sensitive to the MEK inhibitors CI-1040 (**D**), left), and NRAS mutant cell lines from the CCLE dataset were more sensitive to the MEK inhibitors AZD6244 (**E**), left), or PD-0325901 (**F**), left) in CCLE data, but all NRAS mutant cell lines showed limited therapeutic effects in response to the MEK inhibitors when PIM1 mRNA expression levels were high (**D–F**), right).

Among the biomarker gene pairs (Table S2), PPP1R13L, EGFR, IL15RA, or PIM1 are acting in the cancer core pathways. Moreover, upregulated mRNA expression of them was associated with decreased sensitivity of BRAF mutant cells to BRAF inhibitors in both CCLE and CGP datasets (Fig. 2C, Fig. 3A–C). The associations between PPP1R13L, IL15RA, PIM1, or EGFR expression and drug sensitivity in BRAF mutant cell lines were observed not only in three different BRAF inhibitors (Fig. 3A–C), but also two MEK inhibitors AZD6244 and PD-0325901 (Figure. S2). Thus, decreasing the expression of PPP1R13L, IL15RA, PIM1, or EGFR could potentially restore the sensitivity of BRAF mutant cells to BRAF or MEK inhibitors in cells expressing high levels of these genes, and drugs targeting them could be an auxiliary therapy for BRAF or MEK inhibitors. When using the DrugBank database to identify drugs that target these four genes, we found LY294002 to inhibit PIM1 and Gefitinib/Erlotinib to inhibit EGFR while no drugs are available to target PPP1R13L or IL15RA. Notably, a recent study by Prahallad *et al*.^21^ demonstrated that feedback activation of EGFR make BRAF mutant melanoma cells resistant to the BRAF inhibitor PLX4032. This suggests that colon cancer patients with BRAF (V600E) mutations, for whom there are currently no targeted treatment options available, might benefit from a combination therapy of BRAF and EGFR inhibitors. The above finding validated our BRAF-EGFR biomarker pair, but little is known about combination therapy targeting BRAF and PIM1.

### Experimentally validating drug combinations for two-drug therapies

**Figure 4:**
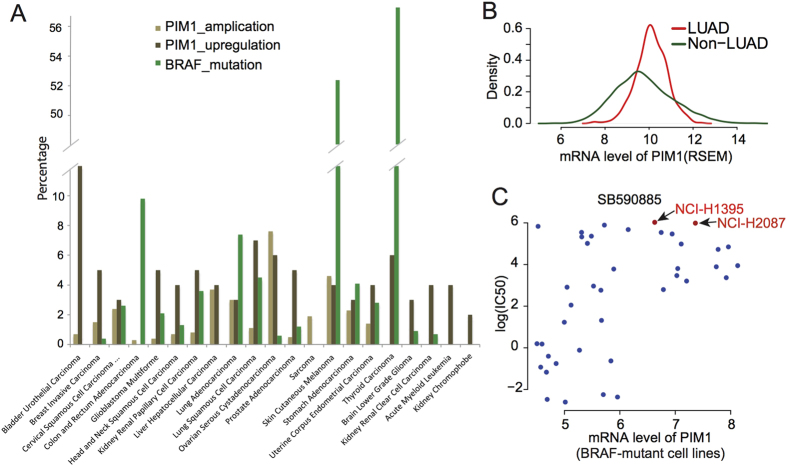
Analysis of the frequency of BRAF mutations and PIM1 amplification or upregulation among different cancer types. (**A**) BRAF mutations and PIM upregulation frequently occur in different types of cancer from TCGA patient samples. (**B**) Analysis of TCGA samples shows that PIM1 mRNA is highly expressed in lung adenocarcinoma (LUAD) tumors relative to non-LUAD tumors. (**C**) Cell lines with a BRAF mutation and high PIM1 expression, including LUAD cell lines NCI-H1395 and NCI-H2087, are less sensitive to the BRAF inhibitor SB590885.

High expression of PIM1 was associated with decreased sensitivity of BRAF mutant cell lines to the MEK inhibitor PD-0325901 (Figure. S2C). Moreover, high expression of PIM1 was associated with decreased sensitivity of NRAS mutant cell lines to MEK inhibitors in both the CCLE and CGP datasets (Fig. 3D–F). NRAS mutation has overlapping functional consequences with BRAF mutation. From The Cancer Genome Atlas (TCGA)^40^, we found that BRAF mutation, PIM1 amplification or upregulation^41^ occur frequently in a wide range of cancers (Fig. 4A). In addition, analysis of expression data of lung adenocarcinoma (LUAD) and 26 non-LUAD tumors in TCGA suggests that LUAD patients tend to express higher levels of PIM1 mRNA (student t test p < 2.2e–6, Fig. 4B). Among the BRAF mutant cell lines we initially studied, two LUAD cell lines, NCI-H1395 and NCI-H2087, had higher PIM1 expression and were less sensitive to the BRAF inhibitor SB590885 (Fig. 4C). So we selected these two cell lines for experimental validation.

**Figure 5:**
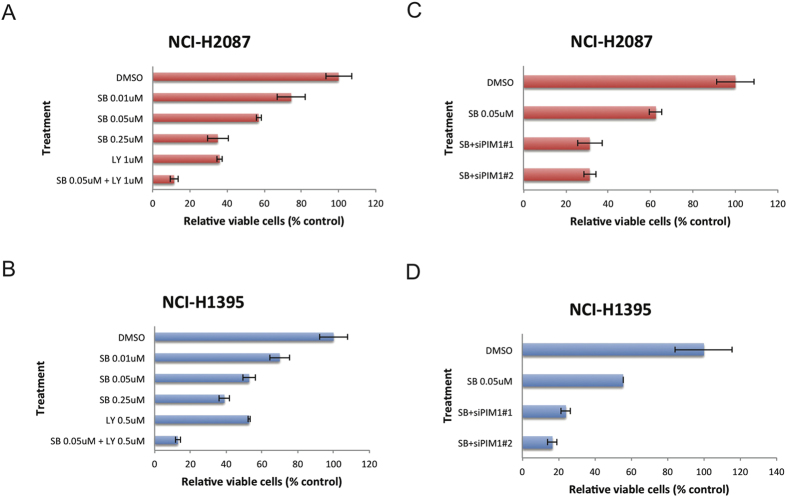
A drug combination inhibits cell proliferation more effectively in lung adenocarcinoma cell lines with a BRAF mutation and PIM1 upregulation. Cell growth inhibition assays for NCI-H2087 (**A**) and NCI-H1395 (**B**) cell lines. Cells were treated with increasing concentrations of SB590885, LY294002, or a combination of SB590885 and LY294002 for 72 h, and cell viability was determined by cell counting using a hemocytometer. Error bars show standard error for the data, and means were derived from two replicates (n = 2). NCI-H2087 (**C**) and NCI-H1395 (**D**) cell lines were transfected with PIM1 siRNA. After six hours, the cell culture media was replaced, SB590885 (0.05 uM) was added to the media, and the cells were cultured for 66 h before they were harvested and counted. Error bars show standard error for the data, and means were derived from two replicates (n = 2).

First, we tested the NCI-H1395 and NCI-H2087 cell lines for their response to either the BRAF inhibitor SB590885 alone or the PIM1 inhibitor LY294002 alone. With increasing drug concentrations, the cell survival rates were inhibited significantly with either drug alone in both cell lines (Figure. S3). Then we tested whether co-treatment with SB590885 and LY294002 resulted in increased sensitivity in the two cell lines. Upon 72 hours of combined treatment of BRAF inhibitor (SB590885) and PIM1 inhibitor (LY294002) for 72 h, cell proliferation was inhibited more calculated using CompuSyn^42^ such that CI values of less than 1, equal to 1, and greater than 1 indicate synergy, additivity, and antagonism, respectively. The CI value for SB590885 and LY294002 combination treatment is 0.143 in NCI-H2087 and 0.077 in NCI-H1395 respectively, indicating strong synergy of co-treatment. We also performed siRNA-mediated knock-down experiments to target PIM1 expression in the NCI-H1395 and NCI-H2087 LUAD cell lines (Fig. 5C,D). siRNA-mediated suppression of PIM1 expression combined with SB590885 treatment resulted in a marked decrease in proliferation of both NCI-H2087 and NCI-H1395 cells relative to treatment with SB590885 alone. These results indicate that inhibition of PIM1 expression could serve as an auxiliary treatment together with BRAF inhibition in patients with a BRAF mutation and PIM1 upregulation.

To demonstrate that a drug-pair will not make worse the normal cell growth, we looked into GTEX project^43^ in an *in silico* manner. We investigated 2917 samples from the different kinds of normal tissues, with 133 lung samples among them. From the normal tissue, we found 23 normal lung samples with low PIM1 expression (RPKM values were below the 25% quartile of all samples) and no BRAF mutation, suggesting that the two inhibitors if on targets would have minimum effect on their growth.

## Discussion

In this study, we use integrated analysis of pharmacogenomic profiling data to identify pairs of molecular biomarkers that may benefit from the use of combination therapies. This study is not trying to compare the method performance on CCLE and CGP. In fact, there are some recent studies showing significant inconsistencies between the two studies datasets^44^. Instead, we are trying to select predictions that are consistent between CCLE and CGP to ensure the robustness of our finding for experimental validation. Our further analysis showed low consistency between two datasets in terms of the gene pairs as candidates for a two-drug combination therapy (Table S2). There are 200 gene pairs detected by CGP dataset, and 182 gene pairs detected by CCLE dataset. 15 pairs (~8%) appeared in both datasets. BRAF-PIM1, BRAF-EGFR, BRAF-PPP1R13L and BRAF-IL15RA pairs were four of them. Here we preferred to select the most potential gene pairs for experimental validation. BRAF gene is frequently mutated in a wide range of tumors, particularly in skin cutaneous melanoma and thyroid carcinoma (Fig. 4A), and activating mutations in BRAF lead to continuous stimulation of the ERK signaling pathway^45^. BRAF V600E mutation is associated with increased sensitivity to both BRAF inhibitors and MEK inhibitors^5^,^46^,^47^,^48^,^49^. BRAF mutant cells often harbor aberrations in other cancer signaling pathways, and our analysis suggests that other genes may also contribute to the sensitivity of BRAF mutant cells to BRAF inhibitors and MEK inhibitors. Specifically, our integrated analysis predicted that upregulated mRNA expression of EGFR, PPP1R13L, IL15RA, or PIM1 are associated with decreased sensitivity of BRAF mutant cells to BRAF inhibitors. EGFR is a cell surface receptor that binds the EGF ligand. EGF binding to the EGFR induces receptor dimerization and tyrosine autophosphorylation, leading to downstream activation of the PI3K and MAPK signaling pathways^50^. A recent study reported strong synergistic effects when both the EGFR and BRAF are inhibited in colon cancer^21^, a finding consistent with our predictions. PP1R13L is a vital component of the p53 regulatory pathway. It is one of the most evolutionarily conserved negative regulator of p53, and high PPP1R13L expression leads to decreased p53 activity and impaired p53 pathway activity^51^. IL15RA encodes a cytokine receptor that specifically binds interleukin 15 with high affinity and functions in the Jak-STAT signaling pathway. The IL15RA receptor has been reported to enhance cell proliferation and expression of the apoptosis inhibitors BCL2L1/BCL2-XL and BCL2. Increased expression of IL15RA leads to enhanced signaling of the Jak-STAT pathway and decreased apoptosis signaling^52^,^53^. PIM1 is an oncogene that encodes a serine/threonine kinase^54^, which functions in the JAK-STAT signaling pathway regulating cell growth and proliferation. The results of our experimental validation on the BRAF and PIM1 inhibitor combination show that simultaneously controlling the activation of ERK and JAK-STAT signaling is more effective than using a single drug to target one pathway.

We also carried out a large-scale analysis to evaluate how well the detected drug pairs perform on the manually selected pairs of one genomic alternation and one gene expression abnormality with a two-steps investigation. First, we investigated the drug response of cells with a genomic alteration at 52 of the most frequently mutated cancer genes except BRAF. The results showed that cells with BRAF mutations were statistically more sensitive to the BRAF inhibitor SB590885 than cells with other mutated cancer genes (Figure. S4A). Second, we investigated the drug response of cells associated with the gene expression level for 1240 genes functioning in the cancer core pathways. The results showed that the Pearson correlation coefficient values for comparisons between gene expression and drug sensitivity in BRAF mutant cell lines were around 0, indicating most of genes were not associated with drug response at expression level. However, the Pearson correlation values of four potential genes, PPP1R13L, IL15RA, PIM1, and EGFR, were above the 95% quartile of the distribution for 1240 genes (Figure. S4B). Upregulated mRNA expression of these genes was associated with decreased sensitivity of BRAF mutant cells. This analysis showed that combination therapies based on the gene pairs selected by our proposed procedure might be much more convincing than manually selected gene pairs.

Drug sensitivity is the result of complex interactions between multiple genes in an individual tumor, and their combinations are often variable between different tumors. Therefore, it is challenging to generate a common mathematic or statistical model to predict the drug response on new samples correctly. Our study aimed at identifying biomarkers related to the drug response itself, rather than at improving prediction performance. Our approach uses decision trees to identify genomic and transcript biomarkers that cooperatively influence drug sensitivity, and can directly help biologists understand the varied response of cancer cells to therapies. The biomarkers close to the root node include genes in which genomic alterations frequently occur in cancers, such as BRAF mutations. Therefore, BRAF was selected as the first gene in the decision tree for BRAF inhibitors and MEK inhibitors. Activating mutations in KRAS and NRAS, which occur less frequently in cancers, also appeared in the decision trees for MEK inhibitors (Fig. 2A, Figure. S1). The biomarkers close to the leaf node are genes with less frequent mutations in tumors, and despite their potential important contribution to drug sensitivity may only be detectable in large sample cohorts. Multiple genes in the same branch traveling from root node to leaf node cooperatively result in the observed drug sensitivity. Transcript biomarkers were connected with specific nodes in the tree, such that the correlation between gene expression and drug sensitivity exists in the samples under the condition of specific genomic alterations. Such local correlations are more biologically meaningful than correlations that exist across all samples, because gene expression is often associated with other factors. Too many genes are likely to be involved in drug sensitivity, so we applied strict criteria to narrow our search and identify the key genes for two-drug combinations. If focusing on the other nodes in the decision tree, our approach could also provide two-drug combination therapies for cells with a less frequent co-occurrence of two genes alterations, or with multi-drug combination therapies. In the latter case, the side effects or toxicity may need to be taken into consideration.

Despite the encouraging results, our approach suffers from the following limitations, which we hope to address in future studies. First, although we have identified and validated some drug combinations based on the CCLE and CGP datasets, a recent study revealed significant discordance between these two datasets^44^. With the availability of more and better quality pharmocogenomic data, our approach will have more reliable findings. Second, our experimental validation on drug combination of BRAF inhibitor and PIM1 inhibitor was evaluated using two lung cancer cell lines. Extended evaluation should be followed for the potential clinical use, such as validation with larger number of cell lines and mouse models. Third, our analysis cannot tease out the tissue of origin influence which does contribute to the varied response to many anti-cancer agents. For example, in non-small cell lung cancers (NSCLCs), several of the critical signalling pathways are solely controlled by EGFR. When the NSCLCs are exposed to EGFR inhibitors, these pathways are turned off and cancer cells undergo apoptosis^55^. EWS-FLI1 rearrangement is characteristic of Ewing’s sarcoma tumours^33^. Despite this limitation, our approach is expected to identify potential combinations if enough cancer cell lines with a common histology are available. Fourth, the decision tree building process relied heavily on p value ranking. When samples were stratified by mutation, the node with smaller samples has limited power to achieve small p value, which might result in false negatives. However, in this particular study, our analysis yielded many potential gene pairs, so our concern is more focused on controlling false positives rather than false negatives. Finally, we generated a list of candidates for drug combinations, but we just experimentally validated only one prediction. Future work to integrate more information, such as molecular networks or pharmacological feature of drugs as well as to use effect size or combining effect size with p value to control false negatives, can refine our approach for the identification of drug combinations with better power and accuracy. In general, our study demonstrates how published high-throughput pharmacogenomic data, cancer core pathway information, and drug information can be effectively integrated to identify potential cooperative biomarkers for combination therapies.

## Methods

### Dataset analysis

The Cancer Cell Line Encyclopedia (CCLE) study resulted in a large-scale, high-throughput pharmacogenomic dataset for 500 human cancer cell lines, including the mutation status of 1,477 genes, DNA copy number variation (CNV) status of 22,419 genes, mRNA expression profiling of 18,927 genes, and pharmacological profiling for 24 anti-cancer drugs^32^. In this dataset, the drug response is represented by an activity area value, which corresponds to the area over the dose-response curve such that a higher value indicates increased sensitivity.

Results obtained using the CCLE dataset were confirmed using an independent dataset generated by the Cancer Genome Project (CGP) at the Wellcome Trust Sanger Institute^33^. The CGP dataset includes DNA sequence for the complete coding exons of 64 commonly mutated cancer genes and genome-wide analysis of their copy number gain and loss, testing for the presence of seven common cancer gene rearrangements, mRNA expression profiling of 14,500 genes, and pharmacological profiling for 130 anti-cancer drugs. In this dataset, the drug response is represented by the natural logarithm of the IC50 value, which corresponds to the half maximal inhibitory concentration of an anti-cancer drug such that a lower value indicates increased sensitivity.

### Identification of cooperative biomarkers

We built a decision tree for each of the anti-cancer drugs tested to identify genomic alterations — including mutations and CNVs — that contribute to drug sensitivity. At each node of the tree, a single gene in which genomic alterations led to the greatest difference in drug sensitivity between cell lines was selected. We used the p value derived from the Mann-Whitney U-test for two groups of cells separated by the status of an altered gene, using either the mutation status or the CNV status of the gene as the splitting criterion. Using this splitting criterion, the gene selected to split the cells at a given node was expected to be more biologically meaningful than if other standard criteria for the decision tree, such as information gain, were used. The growth of a tree was terminated when no additional genes could be identified that split the cells when p < 0.001 was used, or when less than five cells remained in the node. The decision tree algorithm is based on the selection of the best genes for splitting the samples at each node, which may inadvertently exclude other important genes. To overcome this potential deficit, we improved the decision tree algorithm by removing biomarkers from an existing tree and continuing to generate new trees until no biomarkers could be identified in the new trees.

A decision tree is a hierarchical structure on which cells are organized based on their genomic alteration status. Each node in a tree represents a group of samples that harbor the same genomic alterations. By integrating gene expression information with the decision tree analysis, we were able to identify genes whose expression was associated with drug sensitivity in a specific subset of cells defined by an individual path in the decision tree. For each node, we determined the Pearson correlation coefficient values for comparisons between gene expression and drug sensitivity. We employed a Benjamini-Hochberg multiple testing correction with a false discovery rate of 0.05 to identify a candidate list of significant associations. We removed some genes from consideration that lacked annotations in the cancer core pathways, since their association with anti-cancer drug sensitivity is not understood.

### Pair-wise analysis of biomarkers to identify potential combination therapies

We established criteria for the identification of two biomarkers that could be practical for guiding combination therapies. We identified a genomic alteration, either a mutation or a CNV, as the first biomarker, which could serve as the major alteration leading to tumor progression and contributing to sensitivity to drug X. We used altered mRNA expression of a given gene as the second biomarker, which could serve as an additional alteration contributing to sensitivity to drug X. The first biomarker should meet the following criteria: (1) It was first chosen in the decision tree and correlated with increased sensitivity to drug X, since such a genomic alteration usually occurs with a higher frequency in cancers; and (2) it should have been identified previously as a gene that plays a vital role in tumor progression. The CGP project tested 64 commonly mutated cancer genes, most of which were also tested in the CCLE project. Our analysis assumed these 64 commonly mutated cancer genes play vital roles in cancers. More strict criteria were used to identify second biomarkers with biological significance, including: (1) it must function in a cancer core pathway; (2) its high expression correlates with low sensitivity for drug X; (3) it can be inhibited by drug Y according to DrugBank database; and (4) it has preferably been validated using another pharmacogenomic dataset. If the above criteria are met, tumors harboring alterations in both the first and second biomarkers should respond to combination treatment with drug X and drug Y.

### Cell lines and reagents

NCI-H2087 and NCI-H1395 cell lines were purchased from the CCLE^32^. Cells were cultured in McCoy’s 5 A, 1X (lwakata & Grace Modification) with L-glutamine (CORNING) supplemented with 10% fetal bovine serum (HyClone) and grown under 5% CO2. Cells were grown in LY294002 (EMD Millipore, #440202-5MG) when treated with SB-590885 (Sigma Aldrich, #SML 0501).

### Cell growth inhibition assay for one drug

Cultured cells were seeded into 12-well plates. Twenty-four hours after seeding, serial dilutions of SB590885 were added to cells to final drug concentrations ranging from 0.1–10 μM. Cells were then incubated for 72 h, and the cell number was counted. Relative survival in the presence of SB590885 was normalized to that of untreated controls.

### Cell growth inhibition assay for drug combination

Cultured cells were seeded into 12-well plates. Twenty-four hours after seeding, serial dilutions of SB590885 with concentrations ranging from 0.01–0.25 μM were added to cells. At the same time, 1 μM or 0.5 μM LY294002 was added to NCI-H2087 or NCI-H1395 cells, respectively. Cells were incubated for 72 h, and the cell number was counted. As a control, cells were also cultured in the presence of increasing concentrations of SB590885 alone (0.01–0.25 μM).

### RNA interference and cell growth inhibition assay

Transfections with PIM1 siRNA (Sigma) were performed using Lipofectamine RNAiMax (Life Technologies) according to the manufacturer’s instructions, and cells treated with PIM1 siRNA were incubated with 0.05 μM SB-590885. Cells were then grown for 66 h, and the cell number was counted. Cells were also treated with SB-590885 alone under the same conditions.

## Additional Information

**How to cite this article**: Liu, Y. *et al*. An Integrative Pharmacogenomic Approach Identifies Two-drug Combination Therapies for Personalized Cancer Medicine. *Sci. Rep*. **6**, 22120; doi: 10.1038/srep22120 (2016).

## Supplementary Material

Supplementary Information
