# Supplementary material for: An Integrative Pharmacogenomic Approach Identifies Two-drug Combination Therapies for Personalized Cancer Medicine
Source: Sci Rep. 2016 Feb 26;6:22120. doi: 10.1038/srep22120 (PMC4768263; doi:10.1038/srep22120)
Supplement: Supplementary Information [file srep22120-s1.pdf]

An Integrative Pharmacogenomic Approach Identifies Two-drug Combination Therapies for Personalized Cancer Medicine

Yin Liu[1,2,3], Teng Fei[4,5], Xiaoqi Zheng[6], Myles Brown[4,5], Peng Zhang[7*], X. Shirley Liu[3,5*], and Haiyun Wang[1,3*]

Supplementary figure 1. Combined effects of genomic alterations contributing to drug sensitivity for the MEK inhibitor PD-0325901.

Supplementary figure 2. Upregulation of PPP1R13L, IL15RA, PIM1, or EGFR expression was associated with decreased drug sensitivity for the MEK inhibitors AZD6244 and PD-0325901 in BRAF mutant cell lines.

Supplementary figure 3. Viability of NCI-H2087 and NCI-H1395 lung adenocarcinoma (LUAD) cell lines is inhibited significantly with increasing drug dosage.

Supplementary figure 4. The detected drug pairs perform on the manually selected pairs of one genomic alteration and one gene expression abnormality. (A) Cells with BRAF mutations were statistically more sensitive to BRAF inhibitor SB590885 than cells with manual selected genomic alteration at 52 of the most frequently mutated cancer genes except BRAF. (B) In BRAF mutant cell lines, the Pearson correlation coefficient values for comparisons between gene expression of 1240 genes functioning in the cancer core pathways and drug sensitivity for the BRAF inhibitor SB590885 were around 0. The Pearson correlation values of four potential genes, PPP1R13L, IL15RA, PIM1, and EGFR, were above the 95% quartile of the distribution for 1240 genes.

Supplementary figure 1

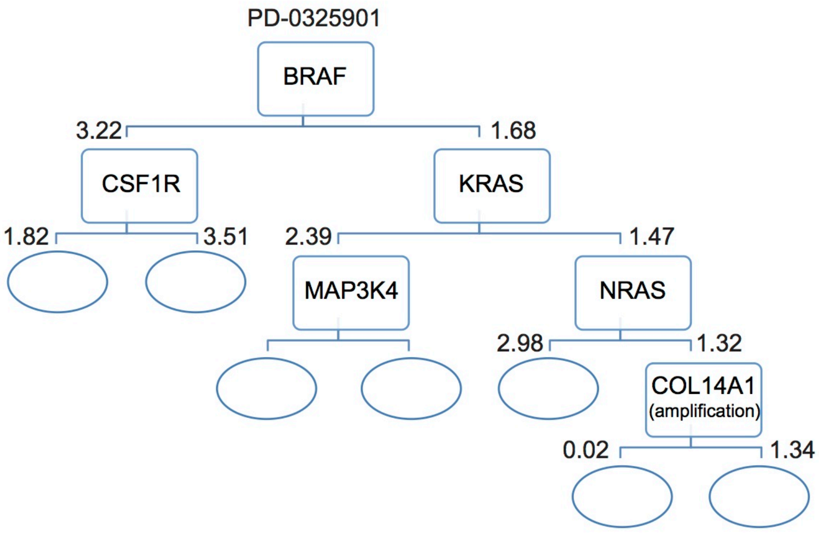

Supplementary figure 2

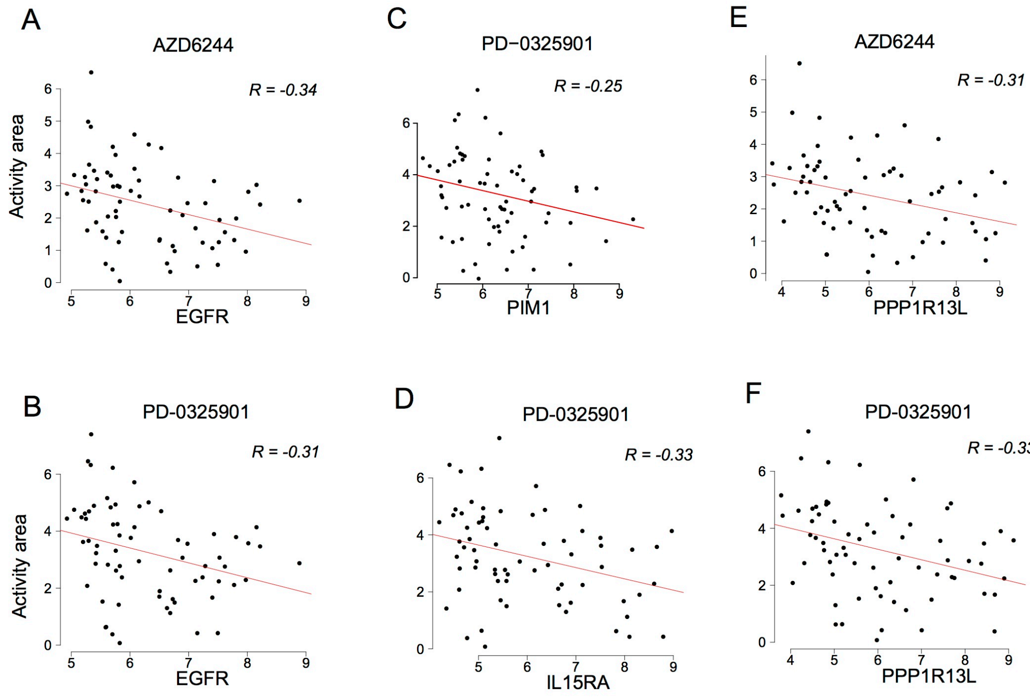

Supplementary figure 3

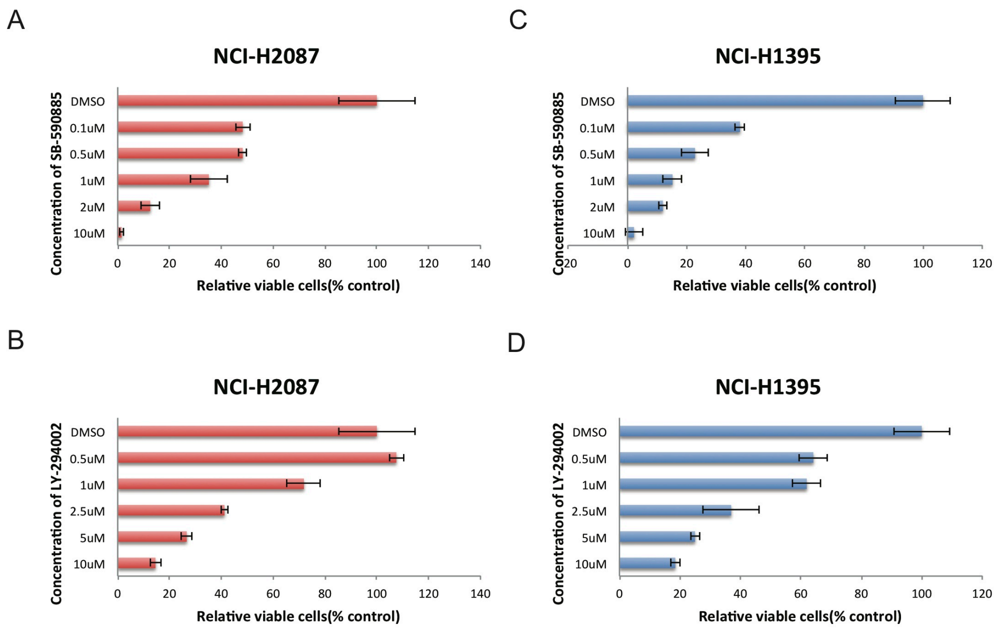

Supplementary figure 4

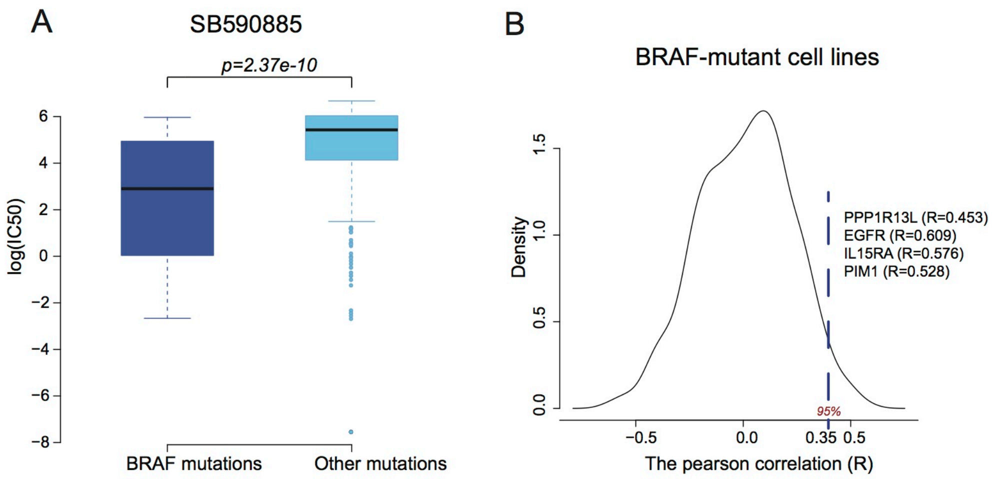

Supplementary table 1. Genes mapped in core cancer pathways annotated by KEGG (Kyoto Encyclopedia of Genes and Genomes), MIPS (The Munich Information Center for Protein Sequences), BIOCARTA, PID (Pathway Interaction Database), and REACTOME databases.

BIOCARTA TGFB PATHWAY	EP300,MAP2K1,APC,MAP3K7,ZFYVE9,TGFB2,TGFB1,CREBBP,MAPK3,TAB1,SMAD3,SMAD4,TGFBR2,SKIL,TGFBR1,SMAD7,TGFB3,CDH1,SMAD2
KEGG TGF BETA SIGNALING PATHWAY	TFDP1,NOG,TNF,GDF7,INHBB,INHBC,COMP,INHBA,THBS4,RHOA,CREBBP,ROCK1,ID1,ID2,RPS6KB1,RPS6KB2,CUL1,LOC728622,ID4,SMAD3,MAPK3,RBL2,SMAD4,RBL1,NODAL,SMAD1,MYC,SMAD2,MAPK1,SMURF2,SMURF1,EP300,BMP8A,GDF5,SKP1,CHRD,TGFB2,TGFB1,IFNG,CDKN2B,PPP2CB,PPP2CA,PPP2R1A,ID3,SMAD5,RBX1,FST,PITX2,PPP2R1B,TGFBR2,AMHR2,LTBP1,LEFTY1,AMH,TGFBR1,SMAD9,LEFTY2,SMAD7,ROCK2,TGFB3,SMAD6,BMPR2,GDF6,BMPR1A,BMPR1B,ACVRL1,ACVR2B,ACVR2A,ACVR1,BMP4,E2F5,BMP2,ACVR1C,E2F4,SP1,BMP7,BMP8B,ZFYVE9,BMP5,BMP6,ZFYVE16,THBS3,INHBE,THBS2,DCN,THBS1,
KEGG WNT SIGNALING PATHWAY	JUN,LRP5,LRP6,PPP3R2,SFRP2,SFRP1,PPP3CC,VANGL1,PPP3R1,FZD1,FZD4,APC2,FZD6,FZD7,SENP2,FZD8,LEF1,CREBBP,FZD9,PRICKLE1,CTBP2,ROCK1,CTBP1,WNT9B,WNT9A,CTNNBIP1,DAAM2,TBL1XR1,MMP7,CER1,MAP3K7,VANGL2,WNT2B,WNT11,WNT10B,DKK2,LOC728622,CHP2,AXIN1,AXIN2,DKK4,NFAT5,MYC,SOX17,CSNK2A1,CSNK2A2,NFATC4,CSNK1A1,NFATC3,CSNK1E,BTRC,PRKX,SKP1,FBXW11,RBX1,CSNK2B,SIAH1,TBL1Y,WNT5B,CCND1,CAMK2A,NLK,CAMK2B,CAMK2D,CAMK2G,PRKACA,APC,PRKACB,PRKACG,WNT16,DAAM1,CHD8,FRAT1,CACYBP,CCND2,NFATC2,NFATC1,CCND3,PLCB2,PLCB1,CSNK1A1L,PRKCB,PLCB3,PRKCA,PLCB4,WIF1,PRICKLE2,PORCN,RHOA,FRAT2,PRKCG,MAPK9,MAPK10,WNT3A,DVL3,RAC2,DVL2,RAC3,FZD3,DKK1,CXXC4,DVL1,FOSL1,CUL1,WNT10A,WNT4,SMAD3,TCF7,SMAD4,RAC1,TCF7L2,SMAD2,WNT1,MAPK8,EP300,WNT7A,GSK3B,WNT7B,PSEN1,WNT8A,WNT8B,WNT2,WNT3,WNT5A,WNT6,CTNNB1,PPP2CB,PPP2CA,PPP2R1A,TBL1X,PPP2R1B,ROCK2,NKD1,FZD10,FZD5,NKD2,TCF7L1,RUVBL1,PPARD,PPP3CB,TP53,PPP3CA,PPP2R5A,PPP2R5E,PPP2R5D,PPP2R5C,PPP2R5B,FZD2,SFRP5,SFRP4,CHP
BIOCARTA WNT PATHWAY	CSNK2A1,WNT1,CSNK1D,CSNK1A1,GSK3B,BTRC,WIF1,CTNNB1,FZD1,PPP2CA,CREBBP,LEF1,TLE1,CCND1,CTBP1,NLK,APC,MAP3K7,DVL1,PPARD,FRAT1,AXIN1,HDAC1,TAB1,SMAD4,MYC,
KEGG HEDGEHOG SIGNALING PATHWAY	CSNK1A1L,HHIP,PTCH2,GAS1,WNT3A,ZIC2,WNT9B,WNT9A,LRP2,CSNK1G1,WNT2B,WNT11,WNT10B,IHH,SMO,WNT10A,WNT4,CSNK1G3,SHH,WNT1,CSNK1D,RAB23,CSNK1A1,CSNK1G2,CSNK1E,BMP8A,GSK3B,WNT7A,BTRC,WNT7B,WNT8A,WNT8B,WNT2,WNT3,PRKX,WNT5A,WNT6,FBXW11,STK36,WNT5B,GLI1,DHH,PRKACA,PRKACB,SUFU,BMP4,PRKACG,BMP2,GLI2,BMP7,GLI3,PTCH1,BMP8B,WNT16,BMP5,BMP6,
PID HIF1APATHWAY	HIF1A,OS9,VHL,HSP90AA1,HIF1AN,TCEB1,NAA10,TCEB2,COPS5,TP53,RBX1,ARNT,EGLN3,EGLN1,CUL2,CDKN2A,GNB2L1,HIF3A,EGLN2
PID TGFBRPATHWAY	EIF2A,PARD6A,NEDD4L,CTNNB1,OCLN,SPTBN1,RPS6KB1,TAB2,TGFB3,CAV1,TGFB1,GRB2,RNF111,PPP2CA,PPP2CB,SMURF1,SMAD7,DAXX,WWP1,TGFB2,DAB2,PDPK1,SOS1,AXIN1,ITCH,RHOA,PPP2R2A,TGFBR1,SMURF2,YWHAE,ZFYVE16,PML,SMAD2,PPP1CA,DACT2,CTGF,STRAP,PPP1R15A,FKBP1A,ARRB2,SMAD4,CAMK2A,SMAD3,DYNLRB1,SKIL,SHC1,MAP3K7,TAB1,XIAP,BAMBI,TGFBRAP1,ZFYVE9,YAP1,TGFBR2,TGFBR3
PID WNT SIGNALING PATHWAY	DKK1,WNT3A,FZD9,WNT2,WNT7A,IGFBP4,WNT1,FZD8,FZD1,KREMEN2,LRP6,KREMEN1,FZD2,LRP5,FZD6,RSPO1,WNT7B,FZD4,CTHRC1,WNT5A,RYK,FZD5,ATP6AP2,WNT3,ROR2,WIF1,FZD10,FZD7
PID WNT CANONICAL PATHWAY	LRP6,GSK3A,AXIN1,CUL3,WNT3A,DVL3,GSK3B,FZD5,PPP2R5A,CSNK1G1,DVL1,PI4K2A,CTNNB1,DVL2,APC,NKD2,PIP5K1B,RANBP3,KLHL12,CAV1,
PID WNT NONCANONICAL PATHWAY	YES1,PRKCZ,MAP3K7,TAB2,FLNA,CSNK1A1,CDC42,DAAM1,FZD7,WNT5A,ROR2,NFATC2,SETDB1,NLK,ROCK1,RAC1,ARRB2,FZD5,MAPK8,CHD7,MAPK9,PPARG,CAMK2A,DVL1,RHOA,FZD6,CTHRC1,DVL2,DVL3,FZD2,TAB1,MAPK10,
PID HEDGEHOG GLIPATHWAY	SUFU,HDAC2,SSPO,PIAS1,GLI2,HDAC1,GNB1,SMO,RAB23,RBBP7,GLI3,CSNK1G3,KIF3A,CREBBP,GLI1,STK36,GNG2,GSK3B,FBXW11,GNAI3,SHH,CSNK1G2,XPO1,CSNK1E,IFT88,CSNK1D,MAP2K1,MTSS1,PRKCD,CSNK1A1,AKT1,GNAZ,IFT172,GNAI1,PTCH1,LGALS3,GNAO1,CSNK1G1,FOXA2,SIN3B,RBBP4,SIN3A,SPOP,ARRB2,GNAI2,SAP30,SAP18,PRKACA

KEGG JAK STAT SIGNALING PATHWAY	STAT3,STAT4,STAT1,STAT2,PIAS3,TYK2,IL21R,CREBBP,SOCS5,IL23A,CTF1,STAM,SPRY4,IL22,JAK1,AKT1,AKT2,JAK3,JAK2,AKT3,STAT5B,STAT5A,TPO,STAT6,MYC,IL24,SPRED1,PIK3R5,CSF2,IL13RA2,PIAS1,IL13RA1,CNTF,PRLR,CNTFR,PRL,SOCS1,IFNE,CSF2RB,CSF2RA,IL23R,CSF3,SOCS3,CCND1,IL22RA2,CSH1,BCL2L1,CSF3R,PIAS4,CBLC,CLCF1,OSMR,IL20,CCND2,CCND3,SOS2,EPO,PTPN11,IL9R,MPL,SPRED2,IFNA5,IFNA4,IFNA2,IL28RA,SOCS2,IFNA1,CISH,PTPN6,IL9,IL7R,IL12RB1,IL12B,IL13,IL12RB2,IL11RA,IL12A,IRF9,IFNA17,PIAS2,IFNA21,IFNA6,LEPR,IFNA7,IFNA8,IFNA10,IFNA13,GRB2,IFNA14,LEP,IFNA16,IL26,IL19,IL10,IL10RA,SOS1,IL10RB,EPOR,IL11,IL3RA,IL3,EP300,IL2RG,IL2RB,CBLB,OSM,CBL,IL15RA,IFNGR2,IL15,IFNG,IFNGR1,IFNB1,IFNAR1,IFNAR2,IL21,IL20RB,IL2RA,IL22RA1,IL20RA,IL2,IL6R,PIK3R3,CRLF2,IFNK,SPRY2,IL6ST,SPRY1,IL7,STAM2,GHR,IL28B,IL29,SOCS4,PIK3CA,PIK3CB,PIM1,IL28A,PIK3CD,LIF,GH1,GH2,IFNW1,LIFR,TSLP,IL4,IL4R,SPRY3,IL5,PIK3CG,SOCS7,IL5RA,PIK3R1,IL6,PIK3R2,
PID NOTCH PATHWAY	NCOR1,NOTCH1,DTX1,FURIN,SSPO,DLL1,DNM1,PTCRA,RAB11A,NEURL,CTBP1,FBXW7,NUMB,CBL,MFAP2,MYCBP,DLL4,MAML1,SPEN,MYC,SKP2,CNTN6,NOTCH2,PSEN1,MAML2,YY1,GATA3,ENO1,ITCH,IL4,APH1B,LNX1,NCOR2,RBPJ,CDKN1A,JAG2,ADAM12,DLK1,EPS15,DNER,SKP1,NOTCH3,RBBP8,NOTCH4,JAG1,APH1A,DLL3,PSENEN,MFAP5,NCSTN,CCND1,EP300,HDAC1,MARK2,MIB1,ADAM10,CUL1,CNTN1,KDM1A,
KEGG NOTCH SIGNALING PATHWAY	HES5,DTX3,NOTCH4,DTX3L,NOTCH3,NOTCH2,EP300,HES1,NOTCH1,NUMB,PSEN2,PSEN1,PTCRA,SNW1,APH1A,KAT2A,ADAM17,RFNG,RBPJ,DTX1,CREBBP,DTX2,MAML1,CTBP2,NCOR2,CTBP1,DVL3,JAG2,DVL2,NUMBL,MAML2,KAT2B,DLL4,PSENEN,DLL3,DVL1,CIR1,DLL1,LFNG,JAG1,MAML3,HDAC1,HDAC2,NCSTN,DTX4,MFNG,RBPJL,
REACTOME G1 S TRANSITION	CDK2,PSMD14,CDK7,CDKN1A,CDKN1B,DBF4,CKS1B,DHFR,E2F1,ORC6,ORC3,POLA2,FBXO5,PPP2R3B,RPA4,MAX,MCM2,MCM3,MCM4,MCM5,MCM6,MCM7,MNAT1,LOC441488,MYC,ORC1,ORC2,ORC4,ORC5,PCNA,POLA1,POLE,POLE2,PPP2CA,PPP2CB,PPP2R1A,PPP2R1B,MCM10,PRIM1,PRIM2,PSMA1,PSMA2,PSMA3,PSMA4,PSMA5,PSMA6,PSMA7,PSMB1,PSMB2,PSMB3,PSMB4,PSMB5,PSMB6,PSMB7,PSMB8,PSMB9,PSMB10,PSMC1,PSMC2,PSMC3,PSMC4,PSMC5,PSMC6,PSMD1,PSMD2,PSMD3,PSMD4,PSMD5,PSMD7,PSMD8,PSMD9,PSMD10,PSMD11,PSMD12,PSMD13,PSME1,PSME2,RB1,RPA1,RPA2,RPA3,RPS27A,RRM2,DHFRP1,LOC645084,SKP1,SKP2,LOC652826,TFDP1,TK2,RPS27AP11,TYMS,LOC729964,UBA52,WEE1,CDT1,CDC7,CDC45,MCM8,CUL1,CCNA2,CCNA1,CCNB1,CCNE1,CCNH,PKMYT1,CCNE2,PSMF1,CDK1,PSMD6,CDC6,CDC25A
REACTOME SIGNALING BY NOTCH	HDAC6,HDAC5,MAMLD1,ADAM10,CDK8,ST3GAL6,TMED2,DTX2,CNTN1,CREBBP,MIB2,JAG1,DTX1,E2F1,E2F3,EIF2C3,EIF2C4,EP300,SNW1,TNRC6B,DTX4,NCSTN,HEY1,HEY2,POFUT1,KAT2A,HEYL,EIF2C1,B4GALT1,EIF2C2,TNRC6A,DLL1,HDAC1,HDAC2,HIF1A,HES1,RBPJ,JAG2,JUN,HES5,LFNG,ARRB1,ARRB2,MFNG,MOV10,LOC441488,MYC,NOTCH2,NOTCH3,NOTCH4,ATP2A1,ATP2A2,ATP2A3,FURIN,APH1A,HDAC7,DLL4,FBXW7,MAML3,PSENEN,HDAC8,PSEN1,PSEN2,POGLUT1,MIB1,TNRC6C,RAB6A,CCND1,RFNG,RPS27A,SEL1L,ST3GAL3,SKP1,ADAM17,TBL1X,TFDP1,TLE1,TLE2,TLE3,TLE4,TP53,LOC728030,RPS27AP11,UBA52,TBL1XR1,HDAC11,APH1B,HDAC10,MAML2,CUL1,NUMB,DLK1,HDAC3,KAT2B,CCNC,NEURL,DNER,NCOR1,NCOR2,HDAC9,HDAC4,MAML1,RBX1
REACTOME SIGNALING BY WNT	FRAT1,PSMD14,FAM123B,PSMA8,CSNK1A1,CTNNB1,PSME4,FRAT2,APC,PPP2CA,PPP2CB,PPP2R1A,PPP2R1B,PPP2R5A,PPP2R5B,PPP2R5C,PPP2R5D,PPP2R5E,PSMA1,PSMA2,PSMA3,PSMA4,PSMA5,PSMA6,PSMA7,PSMB1,PSMB2,PSMB3,PSMB4,PSMB5,PSMB6,PSMB7,PSMB8,PSMB9,PSMB10,PSMC1,PSMC2,PSMC3,PSMC4,PSMC5,PSMC6,PSMD1,PSMD2,PSMD3,PSMD4,PSMD5,PSMD7,PSMD8,PSMD9,PSMD10,PSMD11,PSMD12,PSMD13,PSME1,PSME2,RPS27A,SKP1,LOC652826,RPS27AP11,UBA52,AXIN1,CUL1,BTRC,PSMF1,PSMD6,
REACTOME DOWNREGULATION OF TGF BETA RECEPTOR SIGNALING	STUB1,STRAP,PPP1R15A,SMAD2,SMAD3,SMAD7,UCHL5,PPP1CA,PPP1CB,PPP1CC,PMEPA1,SMURF1,RPS27A,SMURF2,TGFB1,TGFBR1,TGFBR2,RPS27AP11,UBA52,LOC731429,XPO1,MTMR4,ZFYVE9,
REACTOME TGF BETA RECEPTOR SIGNALING IN EMT EPITHELIAL TO MESENCHYMAL TRANSITION	FKBP1A,ARHGEF18,RHOA,F11R,PARD6A,PRKCZ,PARD3,SMURF1,CGN,RPS27A,TGFB1,TGFBR1,TGFBR2,RPS27AP11,UBA52,LOC731429
REACTOME TGF BETA RECEPTOR SIGNALING ACTIVATES SMADS	STUB1,STRAP,FKBP1A,PPP1R15A,SMAD2,SMAD3,SMAD4,SMAD7,FURIN,UCHL5,PPP1CA,PPP1CB,PPP1CC,PMEPA1,SMURF1,RPS27A,SMURF2,TGFB1,TGFBR1,TGFBR2,RPS27AP11,UBA52,LOC731429,XPO1,MTMR4,ZFYVE9,

REACTOME SIGNALING BY TGF BETA RECEPTOR COMPLEX	CDK8,CDK9,STUB1,CDKN2B,STRAP,PARP1,E2F4,E2F5,FKBP1A,ARHGEF18,PPP1R15A,WWTR1,HDAC1,JUNB,RHOA,SMAD2,SMAD3,SMAD4,SMAD7,MEN1,LOC441488,MYC,FURIN,SERPINE1,F11R,PARD6A,UCHL5,TRIM33,PPM1A,PPP1CA,PPP1CB,PPP1CC,PRKCZ,PARD3,PMEPA1,SMURF1,CGN,RBL1,TGIF2,RPS27A,SMURF2,SKI,SKIL,SP1,TFDP1,TGFB1,TGFBR1,TGFBR2,TGIF1,RPS27AP11,UBA52,LOC731429,UBE2D1,UBE2D3,XPO1,USP9X,CCNC,CCNT1,CCNT2,MTMR4,ZFYVE9,NCOR1,NCOR2
REACTOME P53 INDEPENDENT G1 S DNA DAMAGE CHECKPOINT	PSMD14,CHEK1,CHEK2,PSMA8,PSME4,ATM,PSMA1,PSMA2,PSMA3,PSMA4,PSMA5,PSMA6,PSMA7,PSMB1,PSMB2,PSMB3,PSMB4,PSMB5,PSMB6,PSMB7,PSMB8,PSMB9,PSMB10,PSMC1,PSMC2,PSMC3,PSMC4,PSMC5,PSMC6,PSMD1,PSMD2,PSMD3,PSMD4,PSMD5,PSMD7,PSMD8,PSMD9,PSMD10,PSMD11,PSMD12,PSMD13,PSME1,PSME2,RPS27A,LOC651610,LOC652826,RPS27AP11,UBA52,PSMF1,PSMD6,CDC25A
REACTOME P53 DEPENDENT G1 DNA DAMAGE RESPONSE	CDK2,PSMD14,CDKN1A,CDKN1B,PSMA8,PSME4,MDM2,ATM,PSMA1,PSMA2,PSMA3,PSMA4,PSMA5,PSMA6,PSMA7,PSMB1,PSMB2,PSMB3,PSMB4,PSMB5,PSMB6,PSMB7,PSMB8,PSMB9,PSMB10,PSMC1,PSMC2,PSMC3,PSMC4,PSMC5,PSMC6,PSMD1,PSMD2,PSMD3,PSMD4,PSMD5,PSMD7,PSMD8,PSMD9,PSMD10,PSMD11,PSMD12,PSMD13,PSME1,PSME2,RPS27A,RFWD2,LOC651610,LOC652826,TP53,RPS27AP11,RFWD2P1,UBA52,CCNE1,CCNE2,PSMF1,PSMD6
REACTOME INHIBITION OF REPLICATION INITIATION OF DAMAGED DNA BY RB1 E2F1	E2F1,POLA2,PPP2R3B,LOC441488,POLA1,PPP2CA,PPP2CB,PPP2R1A,PPP2R1B,PRIM1,PRIM2,RB1,TFDP1
REACTOME G2 M DNA DAMAGE CHECKPOINT	CHEK1,CHEK2,ATM,ATR,LOC648152,LOC651610,LOC651921,WEE1,ATRIP,CCNB1,CDK1,CDC25C
REACTOME DNA REPAIR	RAD50,CDK7,CCNO,MAD2L2,POLD3,ERCC8,ALKBH2,DDB1,DDB2,ERCC1,ERCC2,ERCC3,ERCC4,ERCC5,ERCC6,FANCA,FANCC,FANCD2,FANCE,FANCB,FANCF,FANCG,ALKBH3,FEN1,SMUG1,XRCC6,ZBTB32,UBE2T,GTF2H1,GTF2H2,GTF2H3,GTF2H4,H2AFX,APEX1,LOC389901,LIG1,LIG3,LIG4,MGMT,MNAT1,MPG,MRE11A,MUTYH,NBN,ATM,NTHL1,OGG1,PCNA,REV1,POLB,POLD1,POLD2,POLE,POLE2,POLH,POLR2A,POLR2B,POLR2C,POLR2D,POLR2E,POLR2F,POLR2G,POLR2H,POLR2I,POLR2J,POLR2K,POLR2L,ATR,FANCL,TDP1,PRKDC,XAB2,FANCM,POLD4,RAD23B,RAD51,RAD52,REV3L,RFC2,RFC3,RFC4,RFC5,RPA1,RPA2,RPA3,RPS27A,LOC648152,LOC651610,LOC651921,LOC652672,LOC652857,GTF2H2B,BRCA1,BRCA2,TCEA1,TDG,TP53BP1,RPS27AP11,UBA52,USP1,XPA,XPC,XRCC1,XRCC4,XRCC5,PALB2,C17orf70,BRIP1,MBD4,CCNH,C19orf40,MDC1
REACTOME REGULATION OF APOPTOSIS	PSMD14,PSMA8,DAPK1,DAPK3,DCC,UNC5B,PSME4,DAPK2,APPL1,PAK2,PSMA1,PSMA2,PSMA3,PSMA4,PSMA5,PSMA6,PSMA7,PSMB1,PSMB2,PSMB3,PSMB4,PSMB5,PSMB6,PSMB7,PSMB8,PSMB9,PSMB10,PSMC1,PSMC2,PSMC3,PSMC4,PSMC5,PSMC6,PSMD1,PSMD2,PSMD3,PSMD4,PSMD5,PSMD7,PSMD8,PSMD9,PSMD10,PSMD11,PSMD12,PSMD13,PSME1,PSME2,RPS27A,LOC652826,RPS27AP11,UBA52,ARHGAP10,CASP3,CASP9,UNC5A,PSMF1,MAGED1,PSMD6
REACTOME APOPTOSIS	BCL2L11,DNM1L,BCAP31,PSMD14,ADD1,DYNLL2,PSMA8,CTNNB1,DAPK1,DAPK3,DCC,DFFA,DFFB,DSG1,DSG2,DSG3,DSP,E2F1,AKT1,UNC5B,ACIN1,PSME4,FNTA,DAPK2,APPL1,GAS2,BBC3,DBNL,GSN,GZMB,H1F0,HIST1H1C,HIST1H1D,HIST1H1E,HIST1H1B,HIST1H1A,HMGB1,HMGB2,APAF1,APC,BIRC2,XIAP,FAS,FASLG,KPNA1,KPNB1,LMNA,LMNB1,MAPT,LOC441488,NMT1,OCLN,PAK2,MST4,PKP1,PLEC,PMAIP1,CYCS,PPP3R1,PRKCD,PRKCQ,MAPK8,DIABLO,PSMA1,PSMA2,PSMA3,PSMA4,PSMA5,PSMA6,PSMA7,PSMB1,PSMB2,PSMB3,PSMB4,PSMB5,PSMB6,PSMB7,PSMB8,PSMB9,PSMB10,PSMC1,PSMC2,PSMC3,PSMC4,PSMC5,PSMC6,PSMD1,PSMD2,PSMD3,PSMD4,PSMD5,PSMD7,PSMD8,PSMD9,PSMD10,PSMD11,PSMD12,PSMD13,BAD,PSME1,PSME2,PTK2,BAK1,BAX,BCL2,BCL2L1,ROCK1,RPS27A,SATB1,BID,LOC647859,LOC652460,LOC652826,BMX,SPTAN1,TFDP1,TJP1,TNF,TNFRSF1A,TP53,TRAF2,ROCK1P1,RPS27AP11,UBA52,VIM,YWHAB,ARHGAP10,CASP3,CASP6,CASP7,CASP8,CASP9,STK24,CASP10,DYNLL1,TRADD,RIPK1,TNFSF10,FADD,TNFRSF10B,CFLAR,UNC5A,BMF,TJP2,PSMF1,MAGED1,PSMD6,CDH1
REACTOME INTRINSIC PATHWAY FOR APOPTOSIS	BCL2L11,DYNLL2,E2F1,AKT1,BBC3,GZMB,APAF1,XIAP,LOC441488,NMT1,PMAIP1,CYCS,PPP3R1,MAPK8,DIABLO,BAD,BAK1,BAX,BCL2,BCL2L1,BID,TFDP1,TP53,YWHAB,CASP3,CASP7,CASP8,CASP9,DYNLL1,BMF

KEGG APOPTOSIS	CASP10,CASP9,CASP8,CASP7,CHUK,PRKAR2B,TNF,TNFSF10,BIRC3,XIAP,PPP3R2,PPP3CC,PPP3R1,MYD88,FADD,CFLAR,RIPK1,BAD,IRAK4,BID,BAX,IKBKB,CASP6,IL1A,AKT1,CASP3,AKT2,TNFRSF1A,AKT3,CHP2,ATM,ENDOG,NFKB1,NFKBIA,CAPN2,PIK3R5,IKBKG,CAPN1,IL3RA,IL3,RELA,ENDOD1,APAF1,PRKX,CSF2RB,TNFRSF10A,TRAF2,TNFRSF10D,NGF,TNFRSF10B,TNFRSF10C,MAP3K14,IL1RAP,IL1B,IRAK2,IL1R1,IRAK1,TRADD,PIK3R3,BCL2,BCL2L1,BIRC2,IRAK3,PRKACA,PRKACB,PRKACG,PPP3CB,TP53,PPP3CA,PIK3CA,PIK3CB,FAS,DFFA,CYCS,DFFB,PIK3CD,LOC651610,PRKAR1A,FASLG,PRKAR2A,PRKAR1B,EXOG,PIK3CG,AIFM1,NTRK1,PIK3R1,PIK3R2,CHP
REACTOME PI3K CASCADE	FRS2,THEM4,KLB,DOK1,EIF4B,EIF4E,EIF4EBP1,EIF4G1,AKT2,FGF1,FGF2,FGF3,FGF4,FGF5,FGF6,FGF7,FGF8,FGF9,FGF10,FGFR1,FGFR3,FGFR2,FGFR4,MTOR,GAB1,FGF20,FGF22,GRB2,EEF2K,PIK3R4,INS,INSR,IRS1,PDE3B,PRKAG2,PDPK1,CAB39,PIK3C3,PIK3CA,PIK3CB,PIK3R1,PIK3R2,PRKAG3,TLR9,PPM1A,STRADB,PRKAA1,PRKAA2,PRKAB1,PRKAB2,PRKAG1,RPTOR,TRIB3,RHEB,RPS6,RPS6KB1,MLST8,LOC644462,STK11,TSC1,TSC2,LOC729120,LOC730244,FGF23,CAB39L,IRS2,FGF18,FGF17,STRADA,KL,FGF19,
REACTOME PI3K EVENTS IN ERBB4 SIGNALING	AKT3,CDKN1A,CDKN1B,CHUK,THEM4,CREB1,NRG4,HBEGF,ERBB4,EREG,AKT1,AKT2,FOXO1,FOXO3,PHLPP1,MTOR,RICTOR,GSK3A,NRG1,NR4A1,MDM2,FOXO4,PDPK1,PIK3CA,PIK3R1,BAD,PTEN,TRIB3,RPS6KB2,MLST8,BTC,TSC2,LOC729120,LOC731292,MAPKAP1,CASP9,AKT1S1,NRG2,
REACTOME PI3K EVENTS IN ERBB2 SIGNALING	AKT3,CDKN1A,CDKN1B,CHUK,THEM4,CREB1,NRG4,HBEGF,EGF,EGFR,ERBB2,ERBB3,ERBB4,EREG,AKT1,AKT2,FOXO1,FOXO3,PHLPP1,MTOR,RICTOR,GAB1,GRB2,GSK3A,NRG1,NR4A1,MDM2,FOXO4,PDPK1,PIK3CA,PIK3R1,BAD,PTEN,TRIB3,RPS6KB2,MLST8,BTC,TSC2,LOC729120,LOC731292,MAPKAP1,CASP9,AKT1S1,NRG2,
BIOCARTA PTEN PATHWAY	PTK2,PTEN,PDK2,SHC1,FOXO3,PDPK1,BCAR1,CDKN1B,ILK,AKT1,PIK3CA,ITGB1,FASLG,GRB2,MAPK3,PIK3R1,SOS1,MAPK1
REACTOME NEGATIVE REGULATION OF THE PI3K AKT NETWORK	AKT3,THEM4,AKT1,AKT2,PHLPP1,PTEN,TRIB3,LOC729120,LOC731292
REACTOME PI3K AKT ACTIVATION	AKT3,CDKN1A,CDKN1B,CHUK,THEM4,CREB1,AKT1,AKT2,FOXO1,FOXO3,PHLPP1,MTOR,RICTOR,GSK3A,NR4A1,IRS1,RHOA,MDM2,FOXO4,NGF,NTRK1,PDPK1,PIK3CA,PIK3CB,PIK3R1,PIK3R2,BAD,PTEN,TRIB3,RPS6KB2,MLST8,TSC2,LOC729120,LOC731292,MAPKAP1,CASP9,AKT1S1,IRS2,
REACTOME SIGNALLING TO RAS	MAPK14,SHC2,GRB2,HRAS,KRAS,NGF,NRAS,NTRK1,SHC3,MAPK1,MAPK3,MAPK11,MAPK13,MAP2K1,MAP2K2,RAF1,RALA,RALB,RALGDS,MAPK12,SHC1,SOS1,SRC,YWHAB,MAPKAPK3,MAPKAPK2,CDK1
BIOCARTA RAS PATHWAY	CASP9,HRAS,FOXO4,CHUK,RALA,RAF1,BCL2L1,ELK1,RELA,MAP2K1,RALBP1,RALGDS,AKT1,PIK3CA,RHOA,PLD1,MAPK3,BAD,NFKB1,PIK3CG,RAC1,PIK3R1,CDC42,

KEGG MAPK SIGNALING PATHWAY	JUN,MEF2C,ELK4,ELK1,JUND,GADD45B,ZAK,STMN1,RRAS2,MAP3K5,MAP3K1,MAP3K3,MAP3K4,MAP3K7,MAP3K8,AKT1,AKT2,ARRB2,CD14,ARRB1,NRAS,DUSP16,CHP2,RASGRP3,NFKB2,NFKB1,MYC,NFATC4,MAPK14,FLNC,FLNA,KRAS,FLNB,PRKX,TRAF6,TGFB2,DUSP1,DUSP2,TGFB1,TRAF2,BDNF,TAB2,ECSIT,TGFBR2,DUSP7,TGFBR1,DUSP5,DUSP6,DUSP3,TGFB3,PLA2G4E,DUSP4,CACNG5,CACNG4,NF1,PLA2G12A,NFATC2,RASGRP4,MAP3K2,MAX,DUSP10,FGF9,FGF8,FGF7,FGF6,FGF5,FGF3,FGF4,FGF1,FGF2,PTPN5,FGF21,IL1R2,MAPK9,CACNA2D3,MAPK10,MAPK11,RASGRP2,PLA2G2A,MAP2K2,PLA2G4A,MAP2K3,MECOM,PLA2G5,MAPK13,MAP2K1,FGFR2,MAP2K7,RASA2,MAPK8IP2,RASGRF1,MAP2K5,RASGRF2,FGFR4,MAP2K6,MAPK8IP3,MAP3K6,CASP3,MAP3K12,FGFR3,FGFR1,RASA1,FGF14,RPS6KA2,RPS6KA3,FGF17,FGF16,FGF10,GRB2,FGF11,FGF12,FGF13,PLA2G1B,RPS6KA1,MAPKAPK3,IKBKG,HRAS,CACNG2,FGF23,CACNG3,MKNK2,FGF18,STK4,STK3,MAPK8IP1,MOS,RAP1A,MAPT,RAP1B,PPP3CB,PPP3CA,CACNA1H,CACNA1G,CACNA1I,ATF4,TAB1,FOS,TAOK2,RPS6KA6,PPP3R2,CACNG8,PPP5C,PPP3CC,CACNG6,PPP3R1,CACNG7,MAP2K4,ATF2,PDGFRB, JMJD7-PLA2G4B,MAP4K3,PLA2G6,PLA2G2E,PLA2G10,MAP4K4,RPS6KA5,BRAF,IKBKB,PLA2G4B,MAP3K11,CACNA2D2,IL1A,PLA2G2F,DAXX,AKT3,GADD45G,FGF20,RELB,MAPKAPK5,MAPK12,RELA,GNA12,HSPA8,HSPB1,PTPRR,LAMTOR3,GADD45A,NGF,DDIT3,MAP3K14,TAOK1,PDGFA,FGF22,PDGFB,NLK,PDGFRA,PRKACA,PRKACB,PAK1,PRKACG,CRK,CDC25B,CRKL,MAP3K13,PLA2G2D,CDC42,RASGRP1,CACNA2D1,CACNB1,SRF,CACNB2,SOS2,CACNB3,CACNB4,CHUK,CACNG1,PRKCB,RAF1,PRKCA,TNF,PAK2,MKNK1,PLA2G3,PRKCG,PTPN7,RAPGEF2,HSPA1L,CACNA1A,HSPA1B,RAC2,HSPA2,CACNA1D,CACNA1E,RAC3,CACNA1B,HSPA1A,PLA2G12B,CACNA1C,DUSP8,CACNA1F,CACNA1S,MAP4K1,TNFRSF1A,DUSP9,PPM1A,PPM1B,MAPK3,RPS6KA4,CACNA2D4,HSPA6,MAPK7,FGF19,RAC1,SOS1,MAPK1,DUSP14,MAP4K2,MAPK8,EGFR,MAPKAPK2,EGF,RRAS,TAOK3,GNG12,NTF4,IL1B,MRAS,NTF3,IL1R1,TP53,FAS,NR4A1,PLA2G2C,FASLG,NTRK2,NTRK1,CHP
BIOCARTA MAPK PATHWAY	MEF2C,JUN,MEF2D,MAX,MEF2BNB-MEF2B,CHUK,RAF1,MEF2A,STAT1,SHC1,ELK1,PAK2,CEBPA,MKNK1,MAP2K4,RIPK1,ATF2,RAPGEF2,MAPK9,MAP3K5,MAP4K3,MAPK10,MAPK11,MAP2K2,MAP4K4,BRAF,RPS6KA5,MAP2K3,MAP3K1,MAPK13,MAP3K3,MAP2K1,MAP3K4,MAP2K7,MAP3K10,MAP3K9,IKBKB,MAP2K5,MAP3K11,CREB1,MAP2K6,MAP3K7,MAP3K8,MAP3K12,MAP3K6,MAP4K1,MAP4K5,RPS6KA2,RPS6KA3,DAXX,RPS6KB1,RPS6KB2,GRB2,MAPK3,MAPK4,RPS6KA4,MAPK6,NFKB1,MAPK7,RAC1,NFKBIA,MYC,RPS6KA1,MAPK1,MAPKAPK3,HRAS,MAP4K2,MAPK8,MAPKAPK5,MAPK14,MAPK12,RELA,MAPKAPK2,TGFB2,MKNK2,TGFB1,TRAF2,ARAF,MAP3K14,TGFBR1,TRADD,TGFB3,PAK1,SP1,MAP3K13,FOS,MAP3K2
KEGG CELL CYCLE	CDC16,CDC7,CDC45,GADD45B,DBF4,ANAPC1,CREBBP,MDM2,ABL1,SMC1B,LOC728622,GADD45G,ATM,ATR,ANAPC7,RBL2,ANAPC5,RBL1,MYC,CDC14B,SMC1A,CDC14A,LOC731751,SKP1,TGFB2,TGFB1,GADD45A,STAG1,LOC650621,PLK1,RBX1,STAG2,TGFB3,MCM4,ORC6,CCND1,MAD1L1,MCM3,MCM6,MCM5,YWHAB,CCNA2,MCM7,BUB1,CHEK1,WEE2,MCM2,PTTG1,CDC27,CDC25B,CDC25C,LOC651610,CDC25A,CDC6,CDC20,BUB3,YWHAZ,CCND2,YWHAH,CCNB1,YWHAG,YWHAE,CCNE1,ORC3,CCND3,PTTG2,SFN,E2F1,TFDP1,ZBTB17,CDK1,ESPL1,ANAPC10,RAD21,BUB1B,ANAPC11,RB1,SKP2,CUL1,SMAD3,SMAD4,ANAPC2,TFDP2,PRKDC,MAD2L1,ANAPC4,SMAD2,YWHAQ,CHEK2,CDC23,EP300,GSK3B,CDKN2A,CDKN1C,CDKN1B,CDKN1A,CDKN2D,CCNA1,CDKN2B,CDKN2C,FZR1,SMC3,ANAPC13,PCNA,TTK,PKMYT1,CDK2,CDC26,E2F5,CDK4,WEE1,E2F4,E2F3,TP53,E2F2,ORC1,ORC2,CCNE2,CDK6,ORC4,CCNB2,CDK7,MAD2L2,ORC5,HDAC1,HDAC2,CCNH,CCNB3,
MIPS SWI SNF CHROMATIN REMODELING RELATED BRCA1 COMPLEX	ACTL6A,BRCA1,SMARCA2,SMARCA4,SMARCB1,SMARCC1,SMARCC2,SMARCD2,SMARCE1,ARID1A,ARID1B,
MIPS INO80 CHROMATIN REMODELING COMPLEX	ACTL6A,ACTR5,ACTR8,INO80C,INO80E,INO80D,INO80,MCRS1,NFRKB,RUVBL1,RUVBL2,TCF3,INO80B
MIPS SRCAP ASSOCIATED CHROMATIN REMODELING COMPLEX	ACTL6A,ACTR6,EAF2,H2AFZ,RUVBL1,RUVBL2,SRCAP,VPS72,YEATS4,ZNHIT1
MIPS RNA POLYMERASE II COMPLEX CHROMATIN STRUCTURE MODIFYING	CCNC,CDK8,DRAP1,GTF2B,GTF2E1,GTF2F1,GTF2H1,MED21,PCSK4,POLR2A,SMARCB1,SMARCC1,SMARCC2,TBP,SMARCA2,SMARCA4,SMARCD1,SMARCD2,SMARCD3
MIPS RNA POLYMERASE II COMPLEX CHROMATIN STRUCTURE MODIFYING 1	ACTL6A,CCNC,CDK8,MED21,SMARCB1,SMARCC1,SMARCC2,SMARCE1,SMARCD1,SMARCD2,SMARCD3,

MIPS RNA POLYMERASE II COMPLEX CHROMATIN STRUCTURE MODIFYING 2	CCNC,CDK8,CREBBP,ERCC3,GTF2B,GTF2F1,GTF2H3,MED21,KAT2B,POLR2A,SMARCA2,SMARCA4,SMARCB1
KEGG AMINOACYL TRNA BIOSYNTHESIS	CARS2,DARS2,RARS,SARS,VARS2,YARS2,WARS,AARS,FARSA,HARS,SARS2,RARS2,NARS,LARS2,FARSB,AARS2,YARS,NARS2,GARS,IARS2,KARS,WARS2,TARSL2,LARS,VARS,PARS2,MARS2,CARS,IARS,TARS2,SEPSECS,MTFMT,TARS,DARS,HARS2,PSTK,QARS,EARS2,EPRS,FARS2,MARS,
PID NFKAPPABATYPICALPATHWAY	PIK3CA,NFKB1,CSNK2B,SYK,CSNK2A1,PIK3R1,SSPO,MAPK14,LCK,NFKBIA,RELA,SRC,BCL3,IKBKB,ARRB2,REL,CSNK2A2,
PID NFKAPPABCANONICALPATHWAY	CYLD,UBE2D3,TNFAIP3,NFKBIA,XPO1,MALT1,BIRC2,TRAF6,NOD2,PRKCA,RELA,RAN,TNF,IKBKG,NFKB1,SSPO,IKBKB,RIPK2,BCL10,CHUK,ATM,ERC1,TNFRSF1A,
MIPS TNF ALPHA NF KAPPA B SIGNALING COMPLEX	CHUK,IKBKG,KPNA3,NFKB1,NFKB2,NFKBIA,NFKBIB,NFKBIE,REL,RELA,RELB,TNIP2
MIPS TNF ALPHA NF KAPPA B SIGNALING COMPLEX 1	CHUK,FBXW7,IKBKB,IKBKG,MAP3K14,NFKB2,REL,RELA,SEC16A,USP2
MIPS TNF ALPHA NF KAPPA B SIGNALING COMPLEX 2	ANKRD28,BTRC,CHUK,CUL1,IKBKE,NFKB2,PPP6C,REL,RELA,PPP6R1,PPP6R2,SKP1
MIPS TNF ALPHA NF KAPPA B SIGNALING COMPLEX 3	CHUK,DDX3X,GLG1,GTF2I,IKBKG,MAP3K8,NFKB1,NFKB2,NFKBIA,NFKBIB,REL,RELA,RELB,RPL30,RPL6,RPS13,TNIP2,
MIPS TNF ALPHA NF KAPPA B SIGNALING COMPLEX 5	CD3EAP,CHUK,CUL1,FBXW11,IKBKB,IKBKG,IQGAP2,KPNA2,LRPPRC,MCC,MTIF2,NFKB1,NFKB2,NFKBIB,PDCD2,POLR1A,POLR1B,POLR1D,POLR1E,POLR2H,POLR2L,RASAL2,REL,RELA,SKP1
MIPS TNF ALPHA NF KAPPA B SIGNALING COMPLEX 6	CDC37,CHUK,FBL,HSP90AA1,HSP90AB1,IKBKB,IKBKG,MAP3K14,RPL30,RPL4,RPL6,RPL8,RPS11,RPS13,
MIPS TNF ALPHA NF KAPPA B SIGNALING COMPLEX 10	ATG16L1,CCAR1,CDC37,CHUK,HSP90AA1,HSP90AB1,IKBKG,NFKBIB,TBK1,TXLNA
PID P53REGULATIONPATHWAY	NEDD8,PPM1D,HIPK2,CHEK2,TP53,TP53AIP1,TRIM28,CCNG1,HUWE1,CSNK1E,SETD8,SKP2,ATM,CSE1L,CSNK1D,CSNK1A1,PPP2CA,CDK2,PIN1,CHEK1,SETD7,MDM4,KAT5,CCNA2,SMYD2,E4F1,EP300,PRMT5,KAT2B,PPP1R13L,CSNK1G1,RFWD2,KAT8,DAXX,DYRK2,TTC5,RPL5,RPL11,PRKCD,PPP2R4,MDM2,FBXO11,AKT1,CDKN2A,ATR,ABL1,CSNK1G2,GSK3B,CREBBP,RASSF1,MAPK14,MAPK9,RPL23,USP7,RCHY1,UBE2D1,CSNK1G3,YY1,MAPK8,
KEGG P53 SIGNALING PATHWAY	CASP9,CASP8,SFN,TSC2,IGF1,CDK1,GADD45B,RCHY1,ZMAT3,IGFBP3,RRM2B,BAI1,TP73,SERPINB5,RPRM,PPM1D,BID,MDM4,BAX,MDM2,TP53AIP1,CASP3,TP53I3,PMAIP1,PIDD,RFWD2,PERP,GADD45G,EI24,SESN2,ATM,ATR,SESN3,CHEK2,APAF1,CDKN2A,RRM2,CDKN1A,BBC3,GADD45A,TNFRSF10B,DDB2,GTSE1,SIAH1,CCND1,CD82,PTEN,CDK2,CHEK1,SESN1,CDK4,SERPINE1,TP53,CCNE2,FAS,CDK6,CYCS,STEAP3,CCNB2,LOC651610,CCND2,SHISA5,CCNB1,CCNG1,CCNE1,CCNB3,THBS1,CCNG2,CCND3
BIOCARTA P53HYPOXIA PATHWAY	MAPK8,CSNK1D,ABCB1,CSNK1A1,EP300,HSPA1A,BAX,DNAJB1P1,HIF1A,HIC1,MDM2,TAF1,CDKN1A,AKT1,TP53,NQO1,FHL2,GADD45A,HSP90AA1,IGFBP3,ATM,RPA1,NFKBIB,
BIOCARTA P53 PATHWAY	CCND1,BCL2,E2F1,APAF1,BAX,CDK2,CDK4,MDM2,RB1,CDKN1A,TP53,GADD45A,ATM,TIMP3,PCNA,CCNE1

Supplementary table 2. Potential drug combinations for two-drug therapies.

gene2 expression	correlation	pval	FDR	associated drug	associated drug mechanism	gene1 genomic alteraion	pathway	has Drug	data source
ACVR1	0.81604649	0.00105	0.00468	ZM-447439	AURKB	START->NOTCH1(mutation)	KEGG TGF BETA SIGNALING PATHWAY	yes	CGP
ACVR1C	-0.68428075	0.0072	0.02789	ZD-6474	Abl, EGFR, Flt3, C-KIT, RET, VEGFR-1, KDR, FLT4	START->NLGN4X(deletion)	KEGG TGF BETA SIGNALING PATHWAY	no	CCLE
ATP2A3	0.507696131	0.00665	0.01865	AZ628	BRAF	START->BRAF(mutation)	REACTOME SIGNALING BY NOTCH	no	CGP
ATP2A3	0.332434996	0.00045	0.00233	BMS-536924	IGF1R	START->CDKN2A(mutation)	REACTOME SIGNALING BY NOTCH	no	CGP
ATP2A3	0.473199858	0.00265	0.00955	CI-1040	MEK1/2	START->BRAF(mutation)	REACTOME SIGNALING BY NOTCH	no	CGP
BAMBI	0.336383497	0.00015	0.00089	Sunitinib	PDGFRA, PDGFRB, VEGFR(KDR), KIT, FLT3	START->CDKN2a(p14)(deletion)	PID TGFBRPATHWAY	no	CGP
BCL2L1	-0.79982864	0.001	0.00508	Panobinostat	HDAC	START->IGLL5(deletion)	KEGG JAK STAT SIGNALING PATHWAY,REACTOME APOPTOSIS,REACTOME INTRINSIC PATHWAY FOR APOPTOSIS,KEGG APOPTOSIS,BIOCARTA RAS PATHWAY	yes	CCLE
BCL2L1	-0.41626215	0.0043	0.01839	Panobinostat	HDAC	START->MAP4K4(mutation)	KEGG JAK STAT SIGNALING PATHWAY,REACTOME APOPTOSIS,REACTOME INTRINSIC PATHWAY FOR APOPTOSIS,KEGG APOPTOSIS,BIOCARTA RAS PATHWAY	yes	CCLE
BCL2L1	-0.3559616	0.0022	0.01033	Panobinostat	HDAC	START->BRD2(mutation)	KEGG JAK STAT SIGNALING PATHWAY,REACTOME APOPTOSIS,REACTOME INTRINSIC PATHWAY FOR APOPTOSIS,KEGG APOPTOSIS,BIOCARTA RAS PATHWAY	yes	CCLE
BDNF	-0.33996417	0.002	0.00949	AZD6244	MEK	START->BRAF(mutation)	KEGG MAPK SIGNALING PATHWAY	no	CCLE
BDNF	-0.70129551	0.00435	0.01857	Lapatinib	EGFR, HER2	START->ZPBP2(amplication)	KEGG MAPK SIGNALING PATHWAY	no	CCLE
BDNF	-0.34057326	0.00185	0.00886	PD-0325901	MEK	START->BRAF(mutation)	KEGG MAPK SIGNALING PATHWAY	no	CCLE
BDNF	0.35020081	0.00915	0.02324	SB590885	BRAF	START->BRAF(mutation)	KEGG MAPK SIGNALING PATHWAY	no	CGP
BIRC3	-0.41421565	0.00015	0.00085	AZD6244	MEK	START->BRAF(mutation)	KEGG APOPTOSIS	no	CCLE
BIRC3	-0.66826391	0.0086	0.03181	Lapatinib	EGFR, HER2	START->ZPBP2(amplication)	KEGG APOPTOSIS	no	CCLE
BIRC3	-0.50946028	0.0071	0.02759	LBW242	IAP	START->SOX10(mutation)	KEGG APOPTOSIS	no	CCLE
BIRC3	-0.44012077	0.00005	0.00029	PD-0325901	MEK	START->BRAF(mutation)	KEGG APOPTOSIS	no	CCLE
BIRC3	-0.35791139	0.00075	0.00391	PLX4720	RAF	START->BRAF(mutation)	KEGG APOPTOSIS	no	CCLE
BMP4	0.384243249	0.00465	0.01445	MK-2206	AKT1/2	START->PIK3CA(mutation)	KEGG TGF BETA SIGNALING PATHWAY,KEGG HEDGEHOG SIGNALING PATHWAY	no	CGP
BMP5	-0.76010604	0.00045	0.00242	Lapatinib	EGFR, HER2	START->IKZF3(amplication)	KEGG TGF BETA SIGNALING PATHWAY,KEGG HEDGEHOG SIGNALING PATHWAY	no	CCLE
BMP5	-0.6603267	0.0082	0.03075	Lapatinib	EGFR, HER2	START->ZPBP2(amplication)	KEGG TGF BETA SIGNALING PATHWAY,KEGG HEDGEHOG SIGNALING PATHWAY	no	CCLE
BMP5	-0.65070601	0.00185	0.00886	Lapatinib	EGFR, HER2	START->ERBB2(amplication)	KEGG TGF BETA SIGNALING PATHWAY,KEGG HEDGEHOG SIGNALING PATHWAY	no	CCLE
BMP5	0.338724864	0.00955	0.02394	SB590885	BRAF	START->BRAF(mutation)	KEGG TGF BETA SIGNALING PATHWAY,KEGG HEDGEHOG SIGNALING PATHWAY	no	CGP
BMP7	-0.82641956	0.0002	0.00113	ZD-6474	Abl, EGFR, Flt3, C-KIT, RET, VEGFR-1, KDR, FLT4	START->NLGN4X(deletion)	KEGG TGF BETA SIGNALING PATHWAY,KEGG HEDGEHOG SIGNALING PATHWAY	no	CCLE
BMPR1B	-0.74639567	0.0085	0.03154	ZD-6474	Abl, EGFR, Flt3, C-KIT, RET, VEGFR-1, KDR, FLT4	START->NLGN4X(deletion)	KEGG TGF BETA SIGNALING PATHWAY	no	CCLE
BTC	-0.3032399	0.0034	0.01508	RAF265	Raf kinase B, KDR	START->BRAF(mutation)	REACTOME PI3K EVENTS IN ERBB4 SIGNALING,REACTOME PI3K EVENTS IN ERBB2 SIGNALING	no	CCLE
BUB1	0.305351213	0	0	RDEA119	MEK1/2	START->CDKN2A(mutation)	KEGG CELL CYCLE	no	CGP
CACNA2D3	0.401785224	0.0012	0.0052	AKT_inhibitor_VIII	AKT1/2	START->PIK3CA(mutation)	KEGG MAPK SIGNALING PATHWAY	yes	CGP

CAPN2	0.721214255	0.0098	0.02435	ZM-447439	AURKB	START->NOTCH1(mutation)	KEGG APOPTOSIS	no	CGP
CAV1	0.60941389	0.0048	0.01479	GDC-0449	SMO	START->EWS_FLI1(mutation)	PID TGFBRPATHWAY,PID WNT CANONICAL PATHWAY	no	CGP
CAV1	-0.64991448	0.00885	0.03246	Lapatinib	EGFR, HER2	START->ZPBP2(amplication)	PID TGFBRPATHWAY,PID WNT CANONICAL PATHWAY	no	CCLE
CAV1	0.31345057	0.003	0.01051	Sunitinib	PDGFRA, PDGFRB, VEGFR(KDR), KIT, FLT3	START->CDKN2a(p14)(deletion)	PID TGFBRPATHWAY,PID WNT CANONICAL PATHWAY	no	CGP
CBLB	-0.78873363	0.0034	0.01508	ZD-6474	Abl, EGFR, Flt3, C-KIT, RET, VEGFR-1, KDR, FLT4	START->NLGN4X(deletion)	KEGG JAK STAT SIGNALING PATHWAY	no	CCLE
CBLC	-0.89037798	0.0079	0.02993	Irinotecan	Topoisomerase I	START->TARP(deletion)	KEGG JAK STAT SIGNALING PATHWAY	no	CCLE
CCND1	-0.55958986	0.00375	0.0164	TAE684	ALK	START->HDHD1(deletion)	KEGG WNT SIGNALING PATHWAY,BIOCARTA WNT PATHWAY,KEGG JAK STAT SIGNALING PATHWAY,PID NOTCH PATHWAY,REACTOME SIGNALING BY NOTCH,KEGG CELL CYCLE,KEGG P53 SIGNALING PATHWAY,BIOCARTA P53 PATHWAY	yes	CCLE
CCNE1	0.627121334	0.00315	0.01091	AZD-2281	PARP1/2	START->EWS_FLI1(mutation)	REACTOME G1 S TRANSITION,REACTOME P53 DEPENDENT G1 DNA DAMAGE RESPONSE,KEGG CELL CYCLE,KEGG P53 SIGNALING PATHWAY,BIOCARTA P53 PATHWAY	no	CGP
CD14	-0.38335032	0.00645	0.02556	Panobinostat	HDAC	START->MAP4K4(mutation)	KEGG MAPK SIGNALING PATHWAY	no	CCLE
CD14	0.319057155	0.0033	0.0113	SB590885	BRAF	START->BRAF(mutation)	KEGG MAPK SIGNALING PATHWAY	no	CGP
CDC14B	0.358699063	0.008	0.02117	MK-2206	AKT1/2	START->PIK3CA(mutation)	KEGG CELL CYCLE	no	CGP
CDC14B	0.824688	0.0015	0.00618	ZM-447439	AURKB	START->NOTCH1(mutation)	KEGG CELL CYCLE	no	CGP
CDC6	0.882829208	0	0	ABT-263	BCL2, BCL-XL, BCL-W	START->NOTCH1(mutation)	REACTOME G1 S TRANSITION,KEGG CELL CYCLE	no	CGP
CDH1	0.871516959	0.0042	0.01344	ABT-263	BCL2, BCL-XL, BCL-W	START->NOTCH1(mutation)	BIOCARTA TGFB PATHWAY,REACTOME APOPTOSIS	no	CGP
CDKN1A	-0.68261259	0.00655	0.02589	Panobinostat	HDAC	START->RARA(mutation)	PID NOTCH PATHWAY,REACTOME G1 S TRANSITION,REACTOME P53 DEPENDENT G1 DNA DAMAGE RESPONSE,REACTOME PI3K EVENTS IN ERBB4 SIGNALING,REACTOME PI3K EVENTS IN ERBB2 SIGNALING,REACTOME PI3K AKT ACTIVATION,KEGG CELL CYCLE,KEGG P53 SIGNALING PATHWAY,BIOCARTA P53HYPOXIA PATHWAY,BIOCARTA P53 PATHWAY	no	CCLE
CDKN1C	-0.59304778	0.00755	0.02894	Lapatinib	EGFR, HER2	START->ZPBP2(amplication)	KEGG CELL CYCLE	no	CCLE
CDT1	0.30974866	0.0014	0.00587	MG-132	Proteasome	START->CDKN2A(mutation)	REACTOME G1 S TRANSITION	no	CGP
CDT1	0.301706116	0	0	RDEA119	MEK1/2	START->CDKN2A(mutation)	REACTOME G1 S TRANSITION	no	CGP
CEBPA	0.587964067	0.0075	0.02025	GDC-0449	SMO	START->EWS_FLI1(mutation)	BIOCARTA MAPK PATHWAY	no	CGP
CEBPA	0.773805054	0.00165	0.00662	ZM-447439	AURKB	START->NOTCH1(mutation)	BIOCARTA MAPK PATHWAY	no	CGP
CGN	-0.76429387	0.0066	0.02605	Topotecan	Topoisomerase I	START->IGLL5(deletion)	REACTOME TGF BETA RECEPTOR SIGNALING IN EMT EPITHELIAL TO MESENCHYMAL TRANSITION,REACTOME SIGNALING BY TGF BETA RECEPTOR COMPLEX	no	CCLE
CISH	0.426142597	0.00805	0.02127	CI-1040	MEK1/2	START->BRAF(mutation)	KEGG JAK STAT SIGNALING PATHWAY	no	CGP
CISH	0.403495237	0.0011	0.00486	SB590885	BRAF	START->BRAF(mutation)	KEGG JAK STAT SIGNALING PATHWAY	no	CGP
CSF2RA	-0.79677292	0.00685	0.02684	Topotecan	Topoisomerase I	START->NCRNA00226(deletion)	KEGG JAK STAT SIGNALING PATHWAY	yes	CCLE
CTBP2	0.876060207	0.003	0.01051	ABT-263	BCL2, BCL-XL, BCL-W	START->NOTCH1(mutation)	KEGG WNT SIGNALING PATHWAY,KEGG NOTCH SIGNALING PATHWAY	no	CGP
CTBP2	-0.5208098	0.0077	0.02937	TAE684	ALK	START->HDHD1(deletion)	KEGG WNT SIGNALING PATHWAY,KEGG NOTCH SIGNALING PATHWAY	no	CCLE
CTGF	0.465306728	0.0005	0.00255	AKT_inhibitor_VIII	AKT1/2	START->PIK3CA(mutation)	PID TGFBRPATHWAY	no	CGP
CTGF	0.371992048	0.0092	0.02331	AZD6244	MEK1/2	START->BRAF(mutation)	PID TGFBRPATHWAY	no	CGP

CTGF	-0.56418999	0.00085	0.00438	TAE684	ALK	START->HDHD1(deletion)	PID TGFBRPATHWAY	no	CCLE
CTHRC1	-0.67245035	0.0014	0.00689	Lapatinib	EGFR, HER2	START->ERBB2(amplication)	PID WNT SIGNALING PATHWAY,PID WNT NONCANONICAL PATHWAY	no	CCLE
CTHRC1	-0.63569103	0.0084	0.03128	Lapatinib	EGFR, HER2	START->ZPBP2(amplication)	PID WNT SIGNALING PATHWAY,PID WNT NONCANONICAL PATHWAY	no	CCLE
CTHRC1	-0.63221649	0.00515	0.02131	Lapatinib	EGFR, HER2	START->IKZF3(amplication)	PID WNT SIGNALING PATHWAY,PID WNT NONCANONICAL PATHWAY	no	CCLE
CTHRC1	-0.4316946	0.0093	0.0337	TAE684	ALK	START->HDHD1(deletion)	PID WNT SIGNALING PATHWAY,PID WNT NONCANONICAL PATHWAY	no	CCLE
DAB2	0.510295518	0.00015	0.00089	AKT_inhibitor_VIII	AKT1/2	START->PIK3CA(mutation)	PID TGFBRPATHWAY	no	CGP
DAPK3	-0.3191658	0.00735	0.02835	RAF265	Raf kinase B, KDR	START->BRAF(mutation)	REACTOME REGULATION OF APOPTOSIS,REACTOME APOPTOSIS	yes	CCLE
DAPK3	-0.84501049	0.0014	0.00689	Topotecan	Topoisomerase I	START->NCRNA00226(deletion)	REACTOME REGULATION OF APOPTOSIS,REACTOME APOPTOSIS	yes	CCLE
DCC	-0.74519524	0.0049	0.02047	ZD-6474	Abl, EGFR, Flt3, C-KIT, RET, VEGFR-1, KDR, FLT4	START->NLGN4X(deletion)	REACTOME REGULATION OF APOPTOSIS,REACTOME APOPTOSIS	no	CCLE
DLK1	0.953314631	0.0015	0.00618	Lapatinib	EGFR, ERBB2	START->SMAD4(mutation)	PID NOTCH PATHWAY,REACTOME SIGNALING BY NOTCH	no	CGP
DLL1	-0.32536747	0.0011	0.00555	PLX4720	RAF	START->BRAF(mutation)	PID NOTCH PATHWAY,KEGG NOTCH SIGNALING PATHWAY,REACTOME SIGNALING BY NOTCH	no	CCLE
DLL3	0.803577242	0.0056	0.01651	ABT-263	BCL2, BCL-XL, BCL-W	START->NOTCH1(mutation)	PID NOTCH PATHWAY,KEGG NOTCH SIGNALING PATHWAY	no	CGP
DLL3	-0.53410576	0.0012	0.006	TAE684	ALK	START->HDHD1(deletion)	PID NOTCH PATHWAY,KEGG NOTCH SIGNALING PATHWAY	no	CCLE
DNER	-0.62842692	0.0024	0.01116	Lapatinib	EGFR, HER2	START->IKZF3(amplication)	PID NOTCH PATHWAY,REACTOME SIGNALING BY NOTCH	no	CCLE
DNER	-0.59190681	0.00755	0.02894	Lapatinib	EGFR, HER2	START->ZPBP2(amplication)	PID NOTCH PATHWAY,REACTOME SIGNALING BY NOTCH	no	CCLE
DSG2	0.786307803	0.00015	0.00089	ABT-263	BCL2, BCL-XL, BCL-W	START->NOTCH1(mutation)	REACTOME APOPTOSIS	no	CGP
DSG2	0.312524315	0.00155	0.00633	PF-02341066	MET, ALK	START->CDKN2a(p14)(deletion)	REACTOME APOPTOSIS	no	CGP
DSP	0.87064894	0.00365	0.01217	ABT-263	BCL2, BCL-XL, BCL-W	START->NOTCH1(mutation)	REACTOME APOPTOSIS	no	CGP
DSP	-0.31780542	0.00425	0.01821	PLX4720	RAF	START->BRAF(mutation)	REACTOME APOPTOSIS	no	CCLE
DTX4	0.556636301	0.0044	0.01388	GW_441756	NTRK1	START->STK11(mutation)	KEGG NOTCH SIGNALING PATHWAY,REACTOME SIGNALING BY NOTCH	no	CGP
DUSP1	0.403773657	0.00215	0.00815	AKT_inhibitor_VIII	AKT1/2	START->PIK3CA(mutation)	KEGG MAPK SIGNALING PATHWAY	no	CGP
DUSP1	0.427916315	0.003	0.01051	AZD6244	MEK1/2	START->BRAF(mutation)	KEGG MAPK SIGNALING PATHWAY	no	CGP
DUSP1	-0.33608705	0.0021	0.00991	AZD6244	MEK	START->BRAF(mutation)	KEGG MAPK SIGNALING PATHWAY	no	CCLE
DUSP1	-0.36957179	0.00095	0.00485	PD-0325901	MEK	START->BRAF(mutation)	KEGG MAPK SIGNALING PATHWAY	no	CCLE
DUSP1	-0.34104846	0.00135	0.00667	PLX4720	RAF	START->BRAF(mutation)	KEGG MAPK SIGNALING PATHWAY	no	CCLE
DUSP1	0.507402309	0.00005	0.00032	SB590885	BRAF	START->BRAF(mutation)	KEGG MAPK SIGNALING PATHWAY	no	CGP
DUSP1	-0.86536438	0.0019	0.00907	ZD-6474	Abl, EGFR, Flt3, C-KIT, RET, VEGFR-1, KDR, FLT4	START->NLGN4X(deletion)	KEGG MAPK SIGNALING PATHWAY	no	CCLE
DUSP4	0.941497121	0.00275	0.00983	Lapatinib	EGFR, ERBB2	START->SMAD4(mutation)	KEGG MAPK SIGNALING PATHWAY	no	CGP
DUSP5	0.971346555	0.00145	0.00602	Lapatinib	EGFR, ERBB2	START->SMAD4(mutation)	KEGG MAPK SIGNALING PATHWAY	no	CGP
DUSP5	0.381131502	0.00805	0.02127	PLX4720	BRAF	START->BRAF(mutation)	KEGG MAPK SIGNALING PATHWAY	no	CGP
DUSP6	0.48002835	0.0004	0.0021	AKT_inhibitor_VIII	AKT1/2	START->PIK3CA(mutation)	KEGG MAPK SIGNALING PATHWAY	no	CGP
DUSP6	-0.30702927	0.0094	0.03397	Panobinostat	HDAC	START->BRD2(mutation)	KEGG MAPK SIGNALING PATHWAY	no	CCLE
DUSP9	-0.45608402	0.00955	0.03437	Lapatinib	EGFR, HER2	START->ERBB2(amplication)	KEGG MAPK SIGNALING PATHWAY	no	CCLE

EAF2	0.318342639	0.00085	0.00395	MG-132	Proteasome	START->CDKN2A(mutation)	MIPS SRCAP ASSOCIATED CHROMATIN REMODELING COMPLEX	no	CGP
EGF	0.507864952	0.00525	0.01579	GW_441756	NTRK1	START->STK11(mutation)	REACTOME PI3K EVENTS IN ERBB2 SIGNALING,KEGG MAPK SIGNALING PATHWAY	yes	CGP
EGFR	0.468007362	0.0013	0.00554	AZD6244	MEK1/2	START->BRAF(mutation)	REACTOME PI3K EVENTS IN ERBB2 SIGNALING,KEGG MAPK SIGNALING PATHWAY	yes	CGP
EGFR	-0.3397728	0.0019	0.00907	AZD6244	MEK	START->BRAF(mutation)	REACTOME PI3K EVENTS IN ERBB2 SIGNALING,KEGG MAPK SIGNALING PATHWAY	yes	CCLE
EGFR	-0.31294745	0.0043	0.01839	PD-0325901	MEK	START->BRAF(mutation)	REACTOME PI3K EVENTS IN ERBB2 SIGNALING,KEGG MAPK SIGNALING PATHWAY	yes	CCLE
EGFR	-0.38408421	0.0001	0.00058	PLX4720	RAF	START->BRAF(mutation)	REACTOME PI3K EVENTS IN ERBB2 SIGNALING,KEGG MAPK SIGNALING PATHWAY	yes	CCLE
EGFR	0.352970231	0.00895	0.02289	RDEA119	MEK1/2	START->BRAF(mutation)	REACTOME PI3K EVENTS IN ERBB2 SIGNALING,KEGG MAPK SIGNALING PATHWAY	yes	CGP
EGFR	0.609447994	0	0	SB590885	BRAF	START->BRAF(mutation)	REACTOME PI3K EVENTS IN ERBB2 SIGNALING,KEGG MAPK SIGNALING PATHWAY	yes	CGP
EGFR	-0.49847358	0.0043	0.01839	TAE684	ALK	START->HDHD1(deletion)	REACTOME PI3K EVENTS IN ERBB2 SIGNALING,KEGG MAPK SIGNALING PATHWAY	yes	CCLE
EGLN3	0.476573008	0.00145	0.00602	AZD6244	MEK1/2	START->BRAF(mutation)	PID HIF1APATHWAY	yes	CGP
EGLN3	0.49725094	0.00195	0.00756	CI-1040	MEK1/2	START->BRAF(mutation)	PID HIF1APATHWAY	yes	CGP
EGLN3	0.376552579	0.00875	0.02253	PLX4720	BRAF	START->BRAF(mutation)	PID HIF1APATHWAY	yes	CGP
EGLN3	0.489859729	0.00005	0.00032	SB590885	BRAF	START->BRAF(mutation)	PID HIF1APATHWAY	yes	CGP
ENDOD1	-0.38041627	0.00125	0.00623	Panobinostat	HDAC	START->BRD2(mutation)	KEGG APOPTOSIS	no	CCLE
ENDOD1	0.339769167	0.0003	0.00164	Sunitinib	PDGFRA, PDGFRB, VEGFR(KDR), KIT, FLT3	START->CDKN2a(p14)(deletion)	KEGG APOPTOSIS	no	CGP
ENDOD1	0.317657602	0.0026	0.00943	VX-680	Aurora A/B/C, FLT3, ABL1, JAK2	START->CDKN2a(p14)(deletion)	KEGG APOPTOSIS	no	CGP
ERBB2	-0.51209165	0	0	Panobinostat	HDAC	START->BRD2(mutation)	REACTOME PI3K EVENTS IN ERBB2 SIGNALING	yes	CCLE
ERBB2	-0.40518199	0.0059	0.0238	Panobinostat	HDAC	START->MAP4K4(mutation)	REACTOME PI3K EVENTS IN ERBB2 SIGNALING	yes	CCLE
ERBB2	-0.32813544	0.00205	0.0097	PLX4720	RAF	START->BRAF(mutation)	REACTOME PI3K EVENTS IN ERBB2 SIGNALING	yes	CCLE
ERBB4	-0.32239503	0.0022	0.01033	AZD6244	MEK	START->BRAF(mutation)	REACTOME PI3K EVENTS IN ERBB4 SIGNALING,REACTOME PI3K EVENTS IN ERBB2 SIGNALING	no	CCLE
ERBB4	0.660318194	0.00375	0.01241	GDC-0449	SMO	START->EWS_FLI1(mutation)	REACTOME PI3K EVENTS IN ERBB4 SIGNALING,REACTOME PI3K EVENTS IN ERBB2 SIGNALING	no	CGP
ERBB4	0.846548031	0.0071	0.01953	Lapatinib	EGFR, ERBB2	START->SMAD4(mutation)	REACTOME PI3K EVENTS IN ERBB4 SIGNALING,REACTOME PI3K EVENTS IN ERBB2 SIGNALING	no	CGP
ERBB4	-0.38149077	0.0002	0.00113	PD-0325901	MEK	START->BRAF(mutation)	REACTOME PI3K EVENTS IN ERBB4 SIGNALING,REACTOME PI3K EVENTS IN ERBB2 SIGNALING	no	CCLE
EREG	0.428736452	0.0055	0.0163	AZ628	BRAF	START->BRAF(mutation)	REACTOME PI3K EVENTS IN ERBB4 SIGNALING,REACTOME PI3K EVENTS IN ERBB2 SIGNALING	no	CGP
EREG	0.538315583	0	0	MK-2206	AKT1/2	START->PIK3CA(mutation)	REACTOME PI3K EVENTS IN ERBB4 SIGNALING,REACTOME PI3K EVENTS IN ERBB2 SIGNALING	no	CGP
EREG	-0.3275589	0.005	0.02081	Panobinostat	HDAC	START->BRD2(mutation)	REACTOME PI3K EVENTS IN ERBB4 SIGNALING,REACTOME PI3K EVENTS IN ERBB2 SIGNALING	no	CCLE
EREG	0.365092083	0.00525	0.01579	SB590885	BRAF	START->BRAF(mutation)	REACTOME PI3K EVENTS IN ERBB4 SIGNALING,REACTOME PI3K EVENTS IN ERBB2 SIGNALING	no	CGP
F11R	0.771821626	0.00755	0.02034	ABT-263	BCL2, BCL-XL, BCL-W	START->NOTCH1(mutation)	REACTOME TGF BETA RECEPTOR SIGNALING IN EMT EPITHELIAL TO MESENCHYMAL TRANSITION,REACTOME SIGNALING BY TGF BETA RECEPTOR COMPLEX	no	CGP
FAS	0.568362811	0.00815	0.02145	AZD-2281	PARP1/2	START->EWS_FLI1(mutation)	REACTOME APOPTOSIS,KEGG APOPTOSIS,KEGG MAPK SIGNALING PATHWAY,KEGG P53 SIGNALING PATHWAY	no	CGP
FGF13	0.663606434	0.0019	0.00741	GDC-0449	SMO	START->EWS_FLI1(mutation)	KEGG MAPK SIGNALING PATHWAY	no	CGP

FGF18	-0.49533285	0.00295	0.01336	Lapatinib	EGFR, HER2	START->ZPBP2(amplication)	REACTOME PI3K CASCADE,KEGG MAPK SIGNALING PATHWAY	no	CCLE
FGF18	-0.32013246	0.0034	0.01508	PD-0325901	MEK	START->BRAF(mutation)	REACTOME PI3K CASCADE,KEGG MAPK SIGNALING PATHWAY	no	CCLE
FGF18	-0.3329659	0.00065	0.00342	PLX4720	RAF	START->BRAF(mutation)	REACTOME PI3K CASCADE,KEGG MAPK SIGNALING PATHWAY	no	CCLE
FGF9	-0.59175085	0.00805	0.03035	Lapatinib	EGFR, HER2	START->ZPBP2(amplication)	REACTOME PI3K CASCADE,KEGG MAPK SIGNALING PATHWAY	no	CCLE
FGFR1	-0.65407406	0.0032	0.01432	Lapatinib	EGFR, HER2	START->ERBB2(amplication)	REACTOME PI3K CASCADE,KEGG MAPK SIGNALING PATHWAY	yes	CCLE
FGFR1	-0.60033003	0.0076	0.02908	Lapatinib	EGFR, HER2	START->IKZF3(amplication)	REACTOME PI3K CASCADE,KEGG MAPK SIGNALING PATHWAY	yes	CCLE
FGFR1	-0.61019731	0.00015	0.00085	TAE684	ALK	START->HDHD1(deletion)	REACTOME PI3K CASCADE,KEGG MAPK SIGNALING PATHWAY	yes	CCLE
FGFR2	0.546387934	0.0019	0.00741	GW_441756	NTRK1	START->STK11(mutation)	REACTOME PI3K CASCADE,KEGG MAPK SIGNALING PATHWAY	yes	CGP
FGFR2	-0.33049928	0.002	0.00949	PD-0325901	MEK	START->BRAF(mutation)	REACTOME PI3K CASCADE,KEGG MAPK SIGNALING PATHWAY	yes	CCLE
FGFR2	-0.38385253	0.00005	0.00029	PLX4720	RAF	START->BRAF(mutation)	REACTOME PI3K CASCADE,KEGG MAPK SIGNALING PATHWAY	yes	CCLE
FGFR3	-0.32269356	0.0021	0.00991	PLX4720	RAF	START->BRAF(mutation)	REACTOME PI3K CASCADE,KEGG MAPK SIGNALING PATHWAY	yes	CCLE
FHL2	0.774158395	0.0086	0.02224	ABT-263	BCL2, BCL-XL, BCL-W	START->NOTCH1(mutation)	BIOCARTA P53HYPOXIA PATHWAY	no	CGP
FHL2	-0.51699634	0.0062	0.02476	TAE684	ALK	START->HDHD1(deletion)	BIOCARTA P53HYPOXIA PATHWAY	no	CCLE
FLNA	0.89355029	0.00745	0.02016	Lapatinib	EGFR, ERBB2	START->SMAD4(mutation)	PID WNT NONCANONICAL PATHWAY,KEGG MAPK SIGNALING PATHWAY	no	CGP
FLNB	0.393465791	0.0057	0.01673	AZD6244	MEK1/2	START->BRAF(mutation)	KEGG MAPK SIGNALING PATHWAY	no	CGP
FLNB	0.503471694	0.00025	0.0014	SB590885	BRAF	START->BRAF(mutation)	KEGG MAPK SIGNALING PATHWAY	no	CGP
FLNC	0.387839752	0.00155	0.00633	SB590885	BRAF	START->BRAF(mutation)	KEGG MAPK SIGNALING PATHWAY	no	CGP
FOS	-0.80819866	0.00505	0.02098	ZD-6474	Abl, EGFR, Flt3, C-KIT, RET, VEGFR-1, KDR, FLT4	START->NLGN4X(deletion)	KEGG MAPK SIGNALING PATHWAY,BIOCARTA MAPK PATHWAY	yes	CCLE
FOSL1	0.476134194	0.0003	0.00164	AKT_inhibitor_VIII	AKT1/2	START->PIK3CA(mutation)	KEGG WNT SIGNALING PATHWAY	no	CGP
FOSL1	0.378708409	0.005	0.01525	MK-2206	AKT1/2	START->PIK3CA(mutation)	KEGG WNT SIGNALING PATHWAY	no	CGP
FOXO1	0.763814435	0.00345	0.01169	ZM-447439	AURKB	START->NOTCH1(mutation)	REACTOME PI3K EVENTS IN ERBB4 SIGNALING,REACTOME PI3K EVENTS IN ERBB2 SIGNALING,REACTOME PI3K AKT ACTIVATION	no	CGP
FST	-0.96841221	0.0085	0.03154	Irinotecan	Topoisomerase I	START->TARP(deletion)	KEGG TGF BETA SIGNALING PATHWAY	yes	CCLE
FZD1	-0.4749168	0.004	0.01732	TAE684	ALK	START->HDHD1(deletion)	KEGG WNT SIGNALING PATHWAY,BIOCARTA WNT PATHWAY,PID WNT SIGNALING PATHWAY	no	CCLE
FZD10	0.484523568	0.0052	0.01569	GW_441756	NTRK1	START->STK11(mutation)	KEGG WNT SIGNALING PATHWAY,PID WNT SIGNALING PATHWAY	no	CGP
FZD2	-0.37107696	0.0005	0.00268	AZD6244	MEK	START->BRAF(mutation)	KEGG WNT SIGNALING PATHWAY,PID WNT SIGNALING PATHWAY,PID WNT NONCANONICAL PATHWAY	no	CCLE
FZD2	-0.79538237	0.0007	0.00367	Lapatinib	EGFR, HER2	START->ZPBP2(amplication)	KEGG WNT SIGNALING PATHWAY,PID WNT SIGNALING PATHWAY,PID WNT NONCANONICAL PATHWAY	no	CCLE
FZD2	-0.68600112	0.0019	0.00907	Lapatinib	EGFR, HER2	START->ERBB2(amplication)	KEGG WNT SIGNALING PATHWAY,PID WNT SIGNALING PATHWAY,PID WNT NONCANONICAL PATHWAY	no	CCLE
FZD2	-0.64613905	0.00505	0.02098	Lapatinib	EGFR, HER2	START->IKZF3(amplication)	KEGG WNT SIGNALING PATHWAY,PID WNT SIGNALING PATHWAY,PID WNT NONCANONICAL PATHWAY	no	CCLE
FZD2	-0.35726316	0.0017	0.00822	PD-0325901	MEK	START->BRAF(mutation)	KEGG WNT SIGNALING PATHWAY,PID WNT SIGNALING PATHWAY,PID WNT NONCANONICAL PATHWAY	no	CCLE
FZD2	-0.31199261	0.00405	0.0175	PLX4720	RAF	START->BRAF(mutation)	KEGG WNT SIGNALING PATHWAY,PID WNT SIGNALING PATHWAY,PID WNT NONCANONICAL PATHWAY	no	CCLE
FZD2	-0.64781975	0.0001	0.00058	TAE684	ALK	START->HDHD1(deletion)	KEGG WNT SIGNALING PATHWAY,PID WNT SIGNALING PATHWAY,PID WNT NONCANONICAL PATHWAY	no	CCLE
FZD2	-0.79720814	0.00465	0.01962	Topotecan	Topoisomerase I	START->IGLL5(deletion)	KEGG WNT SIGNALING PATHWAY,PID WNT SIGNALING PATHWAY,PID WNT NONCANONICAL PATHWAY	no	CCLE
FZD3	0.82058582	0.00535	0.01601	ABT-263	BCL2, BCL-XL, BCL-W	START->NOTCH1(mutation)	KEGG WNT SIGNALING PATHWAY	no	CGP

FZD5	0.379083408	0.00655	0.01845	MK-2206	AKT1/2	START->PIK3CA(mutation)	KEGG WNT SIGNALING PATHWAY,PID WNT SIGNALING PATHWAY,PID WNT CANONICAL PATHWAY,PID WNT NONCANONICAL PATHWAY	no	CGP
FZD5	0.360081016	0.00495	0.01512	SB590885	BRAF	START->BRAF(mutation)	KEGG WNT SIGNALING PATHWAY,PID WNT SIGNALING PATHWAY,PID WNT CANONICAL PATHWAY,PID WNT NONCANONICAL PATHWAY	no	CGP
FZD6	-0.75132116	0	0	Lapatinib	EGFR, HER2	START->ZPBP2(amplication)	KEGG WNT SIGNALING PATHWAY,PID WNT SIGNALING PATHWAY,PID WNT NONCANONICAL PATHWAY	no	CCLE
FZD7	-0.65173703	0.00385	0.01677	Topotecan	Topoisomerase I	START->IGLL5(deletion)	KEGG WNT SIGNALING PATHWAY,PID WNT SIGNALING PATHWAY,PID WNT NONCANONICAL PATHWAY	no	CCLE
GADD45B	0.535905463	0.00305	0.01065	AZ628	BRAF	START->BRAF(mutation)	KEGG MAPK SIGNALING PATHWAY,KEGG CELL CYCLE,KEGG P53 SIGNALING PATHWAY	no	CGP
GADD45B	-0.35877626	0.0012	0.006	AZD6244	MEK	START->BRAF(mutation)	KEGG MAPK SIGNALING PATHWAY,KEGG CELL CYCLE,KEGG P53 SIGNALING PATHWAY	no	CCLE
GADD45B	-0.3429132	0.0024	0.01116	PD-0325901	MEK	START->BRAF(mutation)	KEGG MAPK SIGNALING PATHWAY,KEGG CELL CYCLE,KEGG P53 SIGNALING PATHWAY	no	CCLE
GADD45B	0.353007571	0.008	0.02117	SB590885	BRAF	START->BRAF(mutation)	KEGG MAPK SIGNALING PATHWAY,KEGG CELL CYCLE,KEGG P53 SIGNALING PATHWAY	no	CGP
GADD45G	-0.86265254	0.00765	0.02923	Irinotecan	Topoisomerase I	START->TARP(deletion)	KEGG MAPK SIGNALING PATHWAY,KEGG CELL CYCLE,KEGG P53 SIGNALING PATHWAY	no	CCLE
GADD45G	-0.31039053	0.00085	0.00438	PLX4720	RAF	START->BRAF(mutation)	KEGG MAPK SIGNALING PATHWAY,KEGG CELL CYCLE,KEGG P53 SIGNALING PATHWAY	no	CCLE
GAS1	-0.49955395	0.0069	0.02699	Lapatinib	EGFR, HER2	START->ERBB2(amplication)	KEGG HEDGEHOG SIGNALING PATHWAY	no	CCLE
GATA3	0.624378186	0.00595	0.01725	GDC-0449	SMO	START->EWS_FLI1(mutation)	PID NOTCH PATHWAY	no	CGP
GLI3	0.618737235	0.00745	0.02016	AZD-2281	PARP1/2	START->EWS_FLI1(mutation)	KEGG HEDGEHOG SIGNALING PATHWAY,PID HEDGEHOG GLIPATHWAY	no	CGP
GNAI1	-0.60187535	0.0087	0.03207	Lapatinib	EGFR, HER2	START->ERBB2(amplication)	PID HEDGEHOG GLIPATHWAY	yes	CCLE
GNAI1	-0.47966238	0.00795	0.03007	TAE684	ALK	START->HDHD1(deletion)	PID HEDGEHOG GLIPATHWAY	yes	CCLE
GNAI1	0.562303889	0.0099	0.02452	Vorinostat	HDAC inhibitor Class I, IIa, IIb, IV	START->EWS_FLI1(mutation)	PID HEDGEHOG GLIPATHWAY	yes	CGP
GNG12	0.433345124	0.0014	0.00587	AKT_inhibitor_VIII	AKT1/2	START->PIK3CA(mutation)	KEGG MAPK SIGNALING PATHWAY	no	CGP
GNG12	0.383126489	0.00475	0.01468	MK-2206	AKT1/2	START->PIK3CA(mutation)	KEGG MAPK SIGNALING PATHWAY	no	CGP
GNG12	0.33734841	0	0	PD-0332991	CDK4/6	START->CDKN2a(p14)(mutation)	KEGG MAPK SIGNALING PATHWAY	no	CGP
GNG12	0.348746134	0.0006	0.00297	Sunitinib	PDGFRA, PDGFRB, VEGFR(KDR), KIT, FLT3	START->CDKN2a(p14)(deletion)	KEGG MAPK SIGNALING PATHWAY	no	CGP
GSN	0.634843042	0.00785	0.0209	AZD-2281	PARP1/2	START->EWS_FLI1(mutation)	REACTOME APOPTOSIS	yes	CGP
GSN	0.658670694	0.0028	0.00998	GDC-0449	SMO	START->EWS_FLI1(mutation)	REACTOME APOPTOSIS	yes	CGP
GSN	0.653098558	0.00445	0.01399	Vorinostat	HDAC inhibitor Class I, IIa, IIb, IV	START->EWS_FLI1(mutation)	REACTOME APOPTOSIS	yes	CGP
GTSE1	0.606130573	0.00585	0.01705	GDC-0449	SMO	START->EWS_FLI1(mutation)	KEGG P53 SIGNALING PATHWAY	no	CGP
H1F0	0.303830233	0.0001	0.00061	CHIR-99021	GSK3B	START->CDKN2a(p14)(mutation)	REACTOME APOPTOSIS	no	CGP
H1F0	-0.47740721	0.0016	0.00778	Panobinostat	HDAC	START->MAP4K4(mutation)	REACTOME APOPTOSIS	no	CCLE
H1F0	-0.71945561	0.00605	0.02427	ZD-6474	Abl, EGFR, Flt3, C-KIT, RET, VEGFR-1, KDR, FLT4	START->NLGN4X(deletion)	REACTOME APOPTOSIS	no	CCLE
HBEGF	0.302055719	0.0007	0.00338	Erlotinib	EGFR	START->CDKN2a(p14)(mutation)	REACTOME PI3K EVENTS IN ERBB4 SIGNALING,REACTOME PI3K EVENTS IN ERBB2 SIGNALING	no	CGP
HDAC4	0.706664945	0.001	0.0045	GDC-0449	SMO	START->EWS_FLI1(mutation)	REACTOME SIGNALING BY NOTCH	yes	CGP
HES1	0.670398182	0.00375	0.01241	ABT-263	BCL2, BCL-XL, BCL-W	START->NOTCH1(mutation)	KEGG NOTCH SIGNALING PATHWAY,REACTOME SIGNALING BY NOTCH	no	CGP
HEY1	0.397120968	0.0026	0.00943	AKT_inhibitor_VIII	AKT1/2	START->PIK3CA(mutation)	REACTOME SIGNALING BY NOTCH	no	CGP
HEY1	0.711935116	0.00485	0.01491	ZM-447439	AURKB	START->NOTCH1(mutation)	REACTOME SIGNALING BY NOTCH	no	CGP

HIST1H1E	0.361120086	0	0	Erlotinib	EGFR	START->CDKN2A(mutation)	REACTOME APOPTOSIS	no	CGP
HIST1H1E	0.326661149	0.0003	0.00164	Erlotinib	EGFR	START->CDKN2a(p14)(mutation)	REACTOME APOPTOSIS	no	CGP
HSPA2	-0.46271886	0.0041	0.01767	TAE684	ALK	START->HDHD1(deletion)	KEGG MAPK SIGNALING PATHWAY	no	CCLE
HSPA2	-0.76522826	0.00255	0.01177	ZD-6474	Abl, EGFR, Flt3, C-KIT, RET, VEGFR-1, KDR, FLT4	START->NLGN4X(deletion)	KEGG MAPK SIGNALING PATHWAY	no	CCLE
ID1	0.781346811	0.0092	0.02331	ABT-263	BCL2, BCL-XL, BCL-W	START->NOTCH1(mutation)	KEGG TGF BETA SIGNALING PATHWAY	no	CGP
ID1	0.423269426	0.00315	0.01091	AZD6244	MEK1/2	START->BRAF(mutation)	KEGG TGF BETA SIGNALING PATHWAY	no	CGP
ID1	0.424883516	0.00195	0.00756	MK-2206	AKT1/2	START->PIK3CA(mutation)	KEGG TGF BETA SIGNALING PATHWAY	no	CGP
ID1	0.305862797	0	0	PLX4720	BRAF	START->CDKN2A(mutation)	KEGG TGF BETA SIGNALING PATHWAY	no	CGP
ID1	0.49479113	0.0004	0.0021	SB590885	BRAF	START->BRAF(mutation)	KEGG TGF BETA SIGNALING PATHWAY	no	CGP
ID1	-0.55531993	0.00355	0.01564	TAE684	ALK	START->HDHD1(deletion)	KEGG TGF BETA SIGNALING PATHWAY	no	CCLE
ID3	0.391825468	0.00615	0.01766	AZD6244	MEK1/2	START->BRAF(mutation)	KEGG TGF BETA SIGNALING PATHWAY	no	CGP
ID3	-0.40247216	0.00045	0.00242	AZD6244	MEK	START->BRAF(mutation)	KEGG TGF BETA SIGNALING PATHWAY	no	CCLE
ID3	0.434314817	0.0018	0.0071	MK-2206	AKT1/2	START->PIK3CA(mutation)	KEGG TGF BETA SIGNALING PATHWAY	no	CGP
ID3	-0.54444992	0.0004	0.00217	Panobinostat	HDAC	START->MAP4K4(mutation)	KEGG TGF BETA SIGNALING PATHWAY	no	CCLE
ID3	-0.37537769	0.0011	0.00555	PD-0325901	MEK	START->BRAF(mutation)	KEGG TGF BETA SIGNALING PATHWAY	no	CCLE
ID3	-0.4220406	0.00035	0.00192	PLX4720	RAF	START->BRAF(mutation)	KEGG TGF BETA SIGNALING PATHWAY	no	CCLE
ID3	-0.37179599	0.00275	0.01257	RAF265	Raf kinase B, KDR	START->BRAF(mutation)	KEGG TGF BETA SIGNALING PATHWAY	no	CCLE
ID3	-0.86077491	0.0012	0.006	Topotecan	Topoisomerase I	START->IGLL5(deletion)	KEGG TGF BETA SIGNALING PATHWAY	no	CCLE
ID4	-0.43620134	0.0061	0.02444	TAE684	ALK	START->HDHD1(deletion)	KEGG TGF BETA SIGNALING PATHWAY	no	CCLE
IFNE	-0.38775617	0.0012	0.006	LBW242	IAP	START->SOX10(mutation)	KEGG JAK STAT SIGNALING PATHWAY	no	CCLE
IFNE	-0.31359621	0.0047	0.01979	Panobinostat	HDAC	START->BRD2(mutation)	KEGG JAK STAT SIGNALING PATHWAY	no	CCLE
IFNGR1	0.369363094	0.00685	0.01904	MK-2206	AKT1/2	START->PIK3CA(mutation)	KEGG JAK STAT SIGNALING PATHWAY	yes	CGP
IGFBP4	0.357675218	0.0077	0.02061	SB590885	BRAF	START->BRAF(mutation)	PID WNT SIGNALING PATHWAY	no	CGP
IL13RA1	-0.50908888	0.00735	0.02835	TAE684	ALK	START->HDHD1(deletion)	KEGG JAK STAT SIGNALING PATHWAY	no	CCLE
IL13RA2	0.620580414	0.00175	0.00694	ZM-447439	AURKB	START->NOTCH1(mutation)	KEGG JAK STAT SIGNALING PATHWAY	no	CGP
IL15	0.438840445	0.001	0.0045	AKT_inhibitor_VIII	AKT1/2	START->PIK3CA(mutation)	KEGG JAK STAT SIGNALING PATHWAY	no	CGP
IL15	0.474658087	0.0092	0.02331	GW_441756	NTRK1	START->STK11(mutation)	KEGG JAK STAT SIGNALING PATHWAY	no	CGP
IL15	-0.64790094	0.0085	0.03154	Lapatinib	EGFR, HER2	START->ZPBP2(amplication)	KEGG JAK STAT SIGNALING PATHWAY	no	CCLE
IL15	0.431904941	0.00125	0.00537	MK-2206	AKT1/2	START->PIK3CA(mutation)	KEGG JAK STAT SIGNALING PATHWAY	no	CGP
IL15	0.396779441	0.00185	0.00725	SB590885	BRAF	START->BRAF(mutation)	KEGG JAK STAT SIGNALING PATHWAY	no	CGP
IL15RA	-0.32649039	0.00255	0.01177	PD-0325901	MEK	START->BRAF(mutation)	KEGG JAK STAT SIGNALING PATHWAY	no	CCLE
IL15RA	0.49102271	0.001	0.0045	PLX4720	BRAF	START->BRAF(mutation)	KEGG JAK STAT SIGNALING PATHWAY	no	CGP
IL15RA	-0.39532954	0.0002	0.00113	PLX4720	RAF	START->BRAF(mutation)	KEGG JAK STAT SIGNALING PATHWAY	no	CCLE
IL15RA	0.576332746	0.00005	0.00032	SB590885	BRAF	START->BRAF(mutation)	KEGG JAK STAT SIGNALING PATHWAY	no	CGP

IL1A	0.338420469	0.0088	0.02263	AKT_inhibitor_VIII	AKT1/2	START->PIK3CA(mutation)	KEGG APOPTOSIS,KEGG MAPK SIGNALING PATHWAY	no	CGP
IL1R1	-0.63580099	0.0049	0.02047	Panobinostat	HDAC	START->RARA(mutation)	KEGG APOPTOSIS,KEGG MAPK SIGNALING PATHWAY	yes	CCLE
IL1R1	-0.3962955	0.00045	0.00242	Panobinostat	HDAC	START->BRD2(mutation)	KEGG APOPTOSIS,KEGG MAPK SIGNALING PATHWAY	yes	CCLE
IL1R1	-0.31528923	0.00365	0.01603	PD-0325901	MEK	START->BRAF(mutation)	KEGG APOPTOSIS,KEGG MAPK SIGNALING PATHWAY	yes	CCLE
IL1R1	-0.71245147	0.008	0.03021	ZD-6474	Abl, EGFR, Flt3, C-KIT, RET, VEGFR-1, KDR, FLT4	START->NLGN4X(deletion)	KEGG APOPTOSIS,KEGG MAPK SIGNALING PATHWAY	yes	CCLE
IL1R2	0.376367761	0.0097	0.02419	AZD6244	MEK1/2	START->BRAF(mutation)	KEGG MAPK SIGNALING PATHWAY	no	CGP
IL1R2	0.517114437	0.0015	0.00618	CI-1040	MEK1/2	START->BRAF(mutation)	KEGG MAPK SIGNALING PATHWAY	no	CGP
IL1R2	-0.67008946	0.0067	0.02637	Lapatinib	EGFR, HER2	START->ZPBP2(amplication)	KEGG MAPK SIGNALING PATHWAY	no	CCLE
IL1RAP	0.612206664	0.00575	0.01683	GDC-0449	SMO	START->EWS_FLI1(mutation)	KEGG APOPTOSIS	no	CGP
IL20RB	-0.8086213	0.00945	0.03411	Panobinostat	HDAC	START->IGLL5(deletion)	KEGG JAK STAT SIGNALING PATHWAY	no	CCLE
IL20RB	-0.39575522	0.0004	0.00217	Panobinostat	HDAC	START->BRD2(mutation)	KEGG JAK STAT SIGNALING PATHWAY	no	CCLE
IL23A	-0.66225088	0.00645	0.02556	Lapatinib	EGFR, HER2	START->ZPBP2(amplication)	KEGG JAK STAT SIGNALING PATHWAY	no	CCLE
IL24	0.343148425	0.00815	0.02145	AKT_inhibitor_VIII	AKT1/2	START->PIK3CA(mutation)	KEGG JAK STAT SIGNALING PATHWAY	no	CGP
IL24	0.70584206	0.0013	0.00554	GDC-0449	SMO	START->EWS_FLI1(mutation)	KEGG JAK STAT SIGNALING PATHWAY	no	CGP
IL2RG	0.322945515	0.0006	0.00297	BMS-536924	IGF1R	START->CDKN2A(mutation)	KEGG JAK STAT SIGNALING PATHWAY	yes	CGP
IL4R	0.496790967	0.00995	0.02461	AZ628	BRAF	START->BRAF(mutation)	KEGG JAK STAT SIGNALING PATHWAY	no	CGP
IL4R	0.500528953	0.00035	0.00188	AZD6244	MEK1/2	START->BRAF(mutation)	KEGG JAK STAT SIGNALING PATHWAY	no	CGP
IL4R	0.437816601	0.0053	0.0159	CI-1040	MEK1/2	START->BRAF(mutation)	KEGG JAK STAT SIGNALING PATHWAY	no	CGP
IL4R	0.430381588	0.00325	0.01117	PLX4720	BRAF	START->BRAF(mutation)	KEGG JAK STAT SIGNALING PATHWAY	no	CGP
IL4R	0.543597225	0	0	SB590885	BRAF	START->BRAF(mutation)	KEGG JAK STAT SIGNALING PATHWAY	no	CGP
IL7R	0.312791907	0.001	0.0045	MG-132	Proteasome	START->CDKN2A(mutation)	KEGG JAK STAT SIGNALING PATHWAY	no	CGP
IL7R	0.378227168	0.0049	0.01502	RDEA119	MEK1/2	START->BRAF(mutation)	KEGG JAK STAT SIGNALING PATHWAY	no	CGP
INHBA	-0.34129712	0.00275	0.01257	Panobinostat	HDAC	START->BRD2(mutation)	KEGG TGF BETA SIGNALING PATHWAY	no	CCLE
INHBB	0.527830317	0.0072	0.01971	GW_441756	NTRK1	START->STK11(mutation)	KEGG TGF BETA SIGNALING PATHWAY	no	CGP
INHBB	-0.34275707	0.0029	0.01316	Panobinostat	HDAC	START->BRD2(mutation)	KEGG TGF BETA SIGNALING PATHWAY	no	CCLE
IQGAP2	0.412010781	0.00465	0.01445	AZD6244	MEK1/2	START->BRAF(mutation)	MIPS TNF ALPHA NF KAPPA B SIGNALING COMPLEX 5	no	CGP
IRF9	0.515163809	0.00705	0.01943	AZ628	BRAF	START->BRAF(mutation)	KEGG JAK STAT SIGNALING PATHWAY	no	CGP
IRF9	0.419588266	0.00165	0.00662	RDEA119	MEK1/2	START->BRAF(mutation)	KEGG JAK STAT SIGNALING PATHWAY	no	CGP
IRS1	0.408908704	0.00405	0.0131	AZD6244	MEK1/2	START->BRAF(mutation)	REACTOME PI3K CASCADE,REACTOME PI3K AKT ACTIVATION	yes	CGP
IRS1	0.438166109	0.0022	0.0083	PLX4720	BRAF	START->BRAF(mutation)	REACTOME PI3K CASCADE,REACTOME PI3K AKT ACTIVATION	yes	CGP
IRS1	0.34900137	0.00895	0.02289	RDEA119	MEK1/2	START->BRAF(mutation)	REACTOME PI3K CASCADE,REACTOME PI3K AKT ACTIVATION	yes	CGP
IRS1	0.541118118	0.0001	0.00061	SB590885	BRAF	START->BRAF(mutation)	REACTOME PI3K CASCADE,REACTOME PI3K AKT ACTIVATION	yes	CGP
IRS1	0.309346318	0.0017	0.00678	Sunitinib	PDGFRA, PDGFRB, VEGFR(KDR), KIT, FLT3	START->CDKN2a(p14)(deletion)	REACTOME PI3K CASCADE,REACTOME PI3K AKT ACTIVATION	yes	CGP

IRS2	-0.7832832	0.00095	0.00485	Lapatinib	EGFR, HER2	START->ZPBP2(amplication)	REACTOME PI3K CASCADE,REACTOME PI3K AKT ACTIVATION	no	CCLE
IRS2	-0.78125597	0.0007	0.00367	Lapatinib	EGFR, HER2	START->IKZF3(amplication)	REACTOME PI3K CASCADE,REACTOME PI3K AKT ACTIVATION	no	CCLE
IRS2	-0.68697592	0.00225	0.01054	Lapatinib	EGFR, HER2	START->ERBB2(amplication)	REACTOME PI3K CASCADE,REACTOME PI3K AKT ACTIVATION	no	CCLE
IRS2	-0.79075695	0.0041	0.01767	Panobinostat	HDAC	START->IGLL5(deletion)	REACTOME PI3K CASCADE,REACTOME PI3K AKT ACTIVATION	no	CCLE
JAG2	0.801526587	0.00915	0.02324	ABT-263	BCL2, BCL-XL, BCL-W	START->NOTCH1(mutation)	PID NOTCH PATHWAY,KEGG NOTCH SIGNALING PATHWAY,REACTOME SIGNALING BY NOTCH	no	CGP
JAG2	-0.32963776	0.00165	0.008	PLX4720	RAF	START->BRAF(mutation)	PID NOTCH PATHWAY,KEGG NOTCH SIGNALING PATHWAY,REACTOME SIGNALING BY NOTCH	no	CCLE
JUN	0.371510546	0.00575	0.01683	SB590885	BRAF	START->BRAF(mutation)	KEGG WNT SIGNALING PATHWAY,REACTOME SIGNALING BY NOTCH,KEGG MAPK SIGNALING PATHWAY,BIOCARTA MAPK PATHWAY	yes	CGP
JUN	0.324970935	0.0023	0.0086	VX-680	Aurora A/B/C, FLT3, ABL1, JAK2,	START->CDKN2a(p14)(deletion)	KEGG WNT SIGNALING PATHWAY,REACTOME SIGNALING BY NOTCH,KEGG MAPK SIGNALING PATHWAY,BIOCARTA MAPK PATHWAY	yes	CGP
LCK	0.350411906	0.0002	0.00114	BMS-536924	IGF1R	START->CDKN2A(mutation)	PID NFKAPPABATYPICALPATHWAY	yes	CGP
LEF1	0.323035514	0.00035	0.00188	AZD-0530	SRC, ABL1	START->CDKN2A(mutation)	KEGG WNT SIGNALING PATHWAY,BIOCARTA WNT PATHWAY	no	CGP
LEF1	0.36504929	0.00015	0.00089	BMS-536924	IGF1R	START->CDKN2A(mutation)	KEGG WNT SIGNALING PATHWAY,BIOCARTA WNT PATHWAY	no	CGP
LGALS3	0.619885198	0.00525	0.01579	GDC-0449	SMO	START->EWS_FLI1(mutation)	PID HEDGEHOG GLIPATHWAY	yes	CGP
LGALS3	0.343116364	0	0	PD-0332991	CDK4/6	START->CDKN2a(p14)(mutation)	PID HEDGEHOG GLIPATHWAY	yes	CGP
LIFR	-0.3453042	0.00255	0.01177	Panobinostat	HDAC	START->BRD2(mutation)	KEGG JAK STAT SIGNALING PATHWAY	no	CCLE
LMNA	0.309720317	0.00005	0.00032	PD-0332991	CDK4/6	START->CDKN2a(p14)(mutation)	REACTOME APOPTOSIS	no	CGP
LMNB1	0.300703813	0.00135	0.0057	MG-132	Proteasome	START->CDKN2A(mutation)	REACTOME APOPTOSIS	no	CGP
LRP5	-0.31595943	0.00665	0.02621	Panobinostat	HDAC	START->BRD2(mutation)	KEGG WNT SIGNALING PATHWAY,PID WNT SIGNALING PATHWAY	no	CCLE
MAGED1	0.582042168	0	0	Sunitinib	PDGFRA, PDGFRB, VEGFR(KDR), KIT, FLT3	START->CDKN2a(p14)(deletion)	REACTOME REGULATION OF APOPTOSIS,REACTOME APOPTOSIS	no	CGP
MAGED1	0.302672206	0.0037	0.01229	VX-680	Aurora A/B/C, FLT3, ABL1, JAK2,	START->CDKN2a(p14)(deletion)	REACTOME REGULATION OF APOPTOSIS,REACTOME APOPTOSIS	no	CGP
MAP2K6	0.695059257	0.00405	0.0131	AZD-2281	PARP1/2	START->EWS_FLI1(mutation)	KEGG MAPK SIGNALING PATHWAY,BIOCARTA MAPK PATHWAY	no	CGP
MAP2K6	0.35483986	0.00005	0.00032	Dasatinib	ABL, SRC, KIT, PDGFR	START->CDKN2a(p14)(mutation)	KEGG MAPK SIGNALING PATHWAY,BIOCARTA MAPK PATHWAY	no	CGP
MAP2K6	0.642883086	0.0041	0.01321	GDC-0449	SMO	START->EWS_FLI1(mutation)	KEGG MAPK SIGNALING PATHWAY,BIOCARTA MAPK PATHWAY	no	CGP
MAP2K6	-0.76323315	0.005	0.02081	ZD-6474	Abl, EGFR, Flt3, C-KIT, RET, VEGFR-1, KDR, FLT4	START->NLGN4X(deletion)	KEGG MAPK SIGNALING PATHWAY,BIOCARTA MAPK PATHWAY	no	CCLE
MAP3K14	-0.45689166	0	0	AZD6244	MEK	START->BRAF(mutation)	KEGG APOPTOSIS,KEGG MAPK SIGNALING PATHWAY,BIOCARTA MAPK PATHWAY,MIPS TNF ALPHA NF KAPPA B SIGNALING COMPLEX 1,MIPS TNF ALPHA NF KAPPA B SIGNALING COMPLEX 6	no	CCLE
MAP3K14	-0.80221707	0.0008	0.00415	Lapatinib	EGFR, HER2	START->ZPBP2(amplication)	KEGG APOPTOSIS,KEGG MAPK SIGNALING PATHWAY,BIOCARTA MAPK PATHWAY,MIPS TNF ALPHA NF KAPPA B SIGNALING COMPLEX 1,MIPS TNF ALPHA NF KAPPA B SIGNALING COMPLEX 6	no	CCLE
MAP3K14	-0.47129208	0.00005	0.00029	PD-0325901	MEK	START->BRAF(mutation)	KEGG APOPTOSIS,KEGG MAPK SIGNALING PATHWAY,BIOCARTA MAPK PATHWAY,MIPS TNF ALPHA NF KAPPA B SIGNALING COMPLEX 1,MIPS TNF ALPHA NF KAPPA B SIGNALING COMPLEX 6	no	CCLE
MAP3K14	-0.36107687	0.00075	0.00391	PLX4720	RAF	START->BRAF(mutation)	KEGG APOPTOSIS,KEGG MAPK SIGNALING PATHWAY,BIOCARTA MAPK PATHWAY,MIPS TNF ALPHA NF KAPPA B SIGNALING COMPLEX 1,MIPS TNF ALPHA NF KAPPA B SIGNALING COMPLEX 6	no	CCLE
MAP3K5	0.508223293	0.00845	0.02196	AZ628	BRAF	START->BRAF(mutation)	KEGG MAPK SIGNALING PATHWAY,BIOCARTA MAPK PATHWAY	no	CGP
MAP3K5	0.378637266	0.0045	0.0141	RDEA119	MEK1/2	START->BRAF(mutation)	KEGG MAPK SIGNALING PATHWAY,BIOCARTA MAPK PATHWAY	no	CGP

MAP3K5	0.476111038	0.0006	0.00297	SB590885	BRAF	START->BRAF(mutation)	KEGG MAPK SIGNALING PATHWAY,BIOCARTA MAPK PATHWAY	no	CGP
MAP3K8	-0.37071166	0.0004	0.00217	PLX4720	RAF	START->BRAF(mutation)	KEGG MAPK SIGNALING PATHWAY,BIOCARTA MAPK PATHWAY,MIPS TNF ALPHA NF KAPPA B SIGNALING COMPLEX 3	no	CCLE
MAP3K8	0.375120725	0.00605	0.01746	RDEA119	MEK1/2	START->BRAF(mutation)	KEGG MAPK SIGNALING PATHWAY,BIOCARTA MAPK PATHWAY,MIPS TNF ALPHA NF KAPPA B SIGNALING COMPLEX 3	no	CGP
MAP4K1	0.607515108	0.0067	0.01874	GDC-0449	SMO	START->EWS_FLI1(mutation)	KEGG MAPK SIGNALING PATHWAY,BIOCARTA MAPK PATHWAY	no	CGP
MAP4K1	0.394969689	0	0	MG-132	Proteasome	START->CDKN2A(mutation)	KEGG MAPK SIGNALING PATHWAY,BIOCARTA MAPK PATHWAY	no	CGP
MAPT	-0.78646228	0.00895	0.03273	Topotecan	Topoisomerase I	START->IGLL5(deletion)	REACTOME APOPTOSIS,KEGG MAPK SIGNALING PATHWAY	no	CCLE
MECOM	-0.81364411	0.0006	0.00317	Panobinostat	HDAC	START->RARA(mutation)	KEGG MAPK SIGNALING PATHWAY	no	CCLE
MECOM	0.422905272	0.00375	0.01241	PLX4720	BRAF	START->BRAF(mutation)	KEGG MAPK SIGNALING PATHWAY	no	CGP
MECOM	-0.30927476	0.0038	0.01659	PLX4720	RAF	START->BRAF(mutation)	KEGG MAPK SIGNALING PATHWAY	no	CCLE
MECOM	0.398110285	0.0044	0.01388	SB590885	BRAF	START->BRAF(mutation)	KEGG MAPK SIGNALING PATHWAY	no	CGP
MFAP5	-0.62117071	0.0036	0.01584	Lapatinib	EGFR, HER2	START->ZPBP2(amplication)	PID NOTCH PATHWAY	no	CCLE
MGMT	-0.34431661	0.0018	0.00865	PLX4720	RAF	START->BRAF(mutation)	REACTOME DNA REPAIR	yes	CCLE
NEDD4L	0.319408802	0.0007	0.00338	PF-02341066	MET, ALK	START->CDKN2a(p14)(deletion)	PID TGFBRPATHWAY	no	CGP
NEDD4L	0.311534307	0.00125	0.00537	Sunitinib	PDGFRA, PDGFRB, VEGFR(KDR), KIT, FLT3	START->CDKN2a(p14)(deletion)	PID TGFBRPATHWAY	no	CGP
NEDD4L	0.829387325	0.0014	0.00587	ZM-447439	AURKB	START->NOTCH1(mutation)	PID TGFBRPATHWAY	no	CGP
NFKBIE	0.360580678	0.0065	0.01836	AKT_inhibitor_VIII	AKT1/2	START->PIK3CA(mutation)	MIPS TNF ALPHA NF KAPPA B SIGNALING COMPLEX	no	CGP
NGF	-0.84226268	0.0086	0.03181	Topotecan	Topoisomerase I	START->NCRNA00226(deletion)	KEGG APOPTOSIS,REACTOME PI3K AKT ACTIVATION,REACTOME SIGNALLING TO RAS,KEGG MAPK SIGNALING PATHWAY	yes	CCLE
NQO1	0.373443931	0	0	PD-0332991	CDK4/6	START->CDKN2a(p14)(mutation)	BIOCARTA P53HYPOXIA PATHWAY	yes	CGP
NRG1	-0.3040081	0.00495	0.02064	PD-0325901	MEK	START->BRAF(mutation)	REACTOME PI3K EVENTS IN ERBB4 SIGNALING,REACTOME PI3K EVENTS IN ERBB2 SIGNALING	no	CCLE
NRG1	-0.45074377	0.0026	0.01197	TAE684	ALK	START->HDHD1(deletion)	REACTOME PI3K EVENTS IN ERBB4 SIGNALING,REACTOME PI3K EVENTS IN ERBB2 SIGNALING	no	CCLE
NTF3	-0.30145126	0.0027	0.01237	AZD6244	MEK	START->BRAF(mutation)	KEGG MAPK SIGNALING PATHWAY	no	CCLE
OSMR	0.660767863	0.00505	0.01535	AZD-2281	PARP1/2	START->EWS_FLI1(mutation)	KEGG JAK STAT SIGNALING PATHWAY	no	CGP
OSMR	0.64194463	0.00255	0.0093	GDC-0449	SMO	START->EWS_FLI1(mutation)	KEGG JAK STAT SIGNALING PATHWAY	no	CGP
OSMR	-0.66650232	0.0079	0.02993	Lapatinib	EGFR, HER2	START->ZPBP2(amplication)	KEGG JAK STAT SIGNALING PATHWAY	no	CCLE
OSMR	-0.63535095	0.00585	0.02363	Lapatinib	EGFR, HER2	START->IKZF3(amplication)	KEGG JAK STAT SIGNALING PATHWAY	no	CCLE
PDGFA	0.347956886	0.0092	0.02331	AKT_inhibitor_VIII	AKT1/2	START->PIK3CA(mutation)	KEGG MAPK SIGNALING PATHWAY	no	CGP
PDGFRA	-0.57462953	0.0039	0.01696	Lapatinib	EGFR, HER2	START->IKZF3(amplication)	KEGG MAPK SIGNALING PATHWAY	yes	CCLE
PDGFRA	-0.52917434	0.0024	0.01116	Lapatinib	EGFR, HER2	START->ERBB2(amplication)	KEGG MAPK SIGNALING PATHWAY	yes	CCLE
PERP	0.300012537	0.00005	0.00032	PD-0332991	CDK4/6	START->CDKN2a(p14)(mutation)	KEGG P53 SIGNALING PATHWAY	no	CGP
PHLPP1	0.431550824	0.00185	0.00725	MK-2206	AKT1/2	START->PIK3CA(mutation)	REACTOME PI3K EVENTS IN ERBB4 SIGNALING,REACTOME PI3K EVENTS IN ERBB2 SIGNALING,REACTOME NEGATIVE REGULATION OF THE PI3K AKT NETWORK,REACTOME PI3K AKT ACTIVATION	no	CGP
PIK3CG	-0.60694143	0.00775	0.0295	Lapatinib	EGFR, HER2	START->IKZF3(amplication)	KEGG JAK STAT SIGNALING PATHWAY,KEGG APOPTOSIS,BIOCARTA RAS PATHWAY	yes	CCLE

PIK3R3	0.342967787	0.00025	0.0014	BMS-536924	IGF1R	START->CDKN2A(mutation)	KEGG JAK STAT SIGNALING PATHWAY,KEGG APOPTOSIS	yes	CGP
PIK3R3	0.352306797	0.0001	0.00061	Dasatinib	ABL, SRC, KIT, PDGFR	START->CDKN2a(p14)(mutation)	KEGG JAK STAT SIGNALING PATHWAY,KEGG APOPTOSIS	yes	CGP
PIM1	0.515197853	0.00795	0.0211	AZ628	BRAF	START->BRAF(mutation)	KEGG JAK STAT SIGNALING PATHWAY	yes	CGP
PIM1	0.468046422	0.00065	0.00318	MK-2206	AKT1/2	START->PIK3CA(mutation)	KEGG JAK STAT SIGNALING PATHWAY	yes	CGP
PIM1	0.376247059	0.00815	0.02145	PLX4720	BRAF	START->BRAF(mutation)	KEGG JAK STAT SIGNALING PATHWAY	yes	CGP
PIM1	-0.30850487	0.0038	0.01659	PLX4720	RAF	START->BRAF(mutation)	KEGG JAK STAT SIGNALING PATHWAY	yes	CCLE
PIM1	0.52760033	0.00015	0.00089	SB590885	BRAF	START->BRAF(mutation)	KEGG JAK STAT SIGNALING PATHWAY	yes	CGP
PITX2	-0.3480796	0.0011	0.00555	RAF265	Raf kinase B, KDR	START->BRAF(mutation)	KEGG TGF BETA SIGNALING PATHWAY	no	CCLE
PLA2G10	-0.32249331	0.0017	0.00822	RAF265	Raf kinase B, KDR	START->BRAF(mutation)	KEGG MAPK SIGNALING PATHWAY	no	CCLE
PLA2G2A	-0.79355722	0.0046	0.01945	ZD-6474	Abl, EGFR, Flt3, C-KIT, RET, VEGFR-1, KDR, FLT4	START->NLGN4X(deletion)	KEGG MAPK SIGNALING PATHWAY	yes	CCLE
PLCB4	-0.68948487	0.00615	0.0246	Panobinostat	HDAC	START->RARA(mutation)	KEGG WNT SIGNALING PATHWAY	no	CCLE
PLCB4	-0.65156133	0	0	TAE684	ALK	START->HDHD1(deletion)	KEGG WNT SIGNALING PATHWAY	no	CCLE
PMEPA1	0.303679259	0.0011	0.00486	Sunitinib	PDGFRA, PDGFRB, VEGFR(KDR), KIT, FLT3	START->CDKN2a(p14)(deletion)	REACTOME DOWNREGULATION OF TGF BETA RECEPTOR SIGNALING,REACTOME TGF BETA RECEPTOR SIGNALING ACTIVATES SMADS,REACTOME SIGNALING BY TGF BETA RECEPTOR COMPLEX	no	CGP
PPARG	0.586850752	0.00005	0.00032	MK-2206	AKT1/2	START->PIK3CA(mutation)	PID WNT NONCANONICAL PATHWAY	yes	CGP
PPARG	-0.33034302	0.0049	0.02047	Panobinostat	HDAC	START->BRD2(mutation)	PID WNT NONCANONICAL PATHWAY	yes	CCLE
PPP1R13L	0.601037678	0.00145	0.00602	AZ628	BRAF	START->BRAF(mutation)	PID P53REGULATIONPATHWAY	no	CGP
PPP1R13L	-0.31257914	0.00445	0.01892	AZD6244	MEK	START->BRAF(mutation)	PID P53REGULATIONPATHWAY	no	CCLE
PPP1R13L	-0.48357305	0.0011	0.00555	Panobinostat	HDAC	START->MAP4K4(mutation)	PID P53REGULATIONPATHWAY	no	CCLE
PPP1R13L	-0.33436238	0.0027	0.01237	PD-0325901	MEK	START->BRAF(mutation)	PID P53REGULATIONPATHWAY	no	CCLE
PPP1R13L	0.40726832	0.0059	0.01714	PLX4720	BRAF	START->BRAF(mutation)	PID P53REGULATIONPATHWAY	no	CGP
PPP1R13L	-0.40810765	0.00035	0.00192	PLX4720	RAF	START->BRAF(mutation)	PID P53REGULATIONPATHWAY	no	CCLE
PPP1R13L	0.453190177	0.0006	0.00297	SB590885	BRAF	START->BRAF(mutation)	PID P53REGULATIONPATHWAY	no	CGP
PRKAA2	0.440293743	0.00185	0.00725	AZD6244	MEK1/2	START->BRAF(mutation)	REACTOME PI3K CASCADE	no	CGP
PRKAA2	-0.31589008	0.00355	0.01564	AZD6244	MEK	START->BRAF(mutation)	REACTOME PI3K CASCADE	no	CCLE
PRKAA2	0.433851297	0.00725	0.01981	CI-1040	MEK1/2	START->BRAF(mutation)	REACTOME PI3K CASCADE	no	CGP
PRKAA2	-0.34626358	0.0022	0.01033	Panobinostat	HDAC	START->BRD2(mutation)	REACTOME PI3K CASCADE	no	CCLE
PRKAA2	-0.32701428	0.00345	0.01527	PD-0325901	MEK	START->BRAF(mutation)	REACTOME PI3K CASCADE	no	CCLE
PRKAA2	0.385216186	0.00115	0.00503	SB590885	BRAF	START->BRAF(mutation)	REACTOME PI3K CASCADE	no	CGP
PRKAA2	-0.8642544	0.00195	0.00928	Topotecan	Topoisomerase I	START->IGLL5(deletion)	REACTOME PI3K CASCADE	no	CCLE
PRKAA2	-0.75766084	0.0079	0.02993	ZD-6474	Abl, EGFR, Flt3, C-KIT, RET, VEGFR-1, KDR, FLT4	START->NLGN4X(deletion)	REACTOME PI3K CASCADE	no	CCLE
PRKAG2	0.454877269	0.0014	0.00587	AZD6244	MEK1/2	START->BRAF(mutation)	REACTOME PI3K CASCADE	no	CGP
PRKAG2	0.418280926	0.0043	0.01366	PLX4720	BRAF	START->BRAF(mutation)	REACTOME PI3K CASCADE	no	CGP

PRKAG2	0.389832896	0.0037	0.01229	SB590885	BRAF	START->BRAF(mutation)	REACTOME PI3K CASCADE	no	CGP
PRKAG2	-0.63221644	0.00075	0.00391	TAE684	ALK	START->HDHD1(deletion)	REACTOME PI3K CASCADE	no	CCLE
PRKAR2B	-0.92752191	0.00155	0.00756	Topotecan	Topoisomerase I	START->NCRNA00226(deletion)	KEGG APOPTOSIS	yes	CCLE
PRKCB	0.310601576	0	0	RDEA119	MEK1/2	START->CDKN2A(mutation)	KEGG WNT SIGNALING PATHWAY,KEGG MAPK SIGNALING PATHWAY	yes	CGP
PSMA8	-0.82503759	0.0078	0.02965	Topotecan	Topoisomerase I	START->NCRNA00226(deletion)	REACTOME SIGNALING BY WNT,REACTOME P53 INDEPENDENT G1 S DNA DAMAGE CHECKPOINT,REACTOME P53 DEPENDENT G1 DNA DAMAGE RESPONSE,REACTOME REGULATION OF APOPTOSIS,REACTOME APOPTOSIS	no	CCLE
PSMB10	0.480583051	0.00045	0.00233	SB590885	BRAF	START->BRAF(mutation)	REACTOME G1 S TRANSITION,REACTOME SIGNALING BY WNT,REACTOME P53 INDEPENDENT G1 S DNA DAMAGE CHECKPOINT,REACTOME P53 DEPENDENT G1 DNA DAMAGE RESPONSE,REACTOME REGULATION OF APOPTOSIS,REACTOME APOPTOSIS	no	CGP
PSMB9	0.601286277	0.0098	0.02435	Vorinostat	HDAC inhibitor Class I, IIa, IIb, IV	START->EWS_FLI1(mutation)	REACTOME G1 S TRANSITION,REACTOME SIGNALING BY WNT,REACTOME P53 INDEPENDENT G1 S DNA DAMAGE CHECKPOINT,REACTOME P53 DEPENDENT G1 DNA DAMAGE RESPONSE,REACTOME REGULATION OF APOPTOSIS,REACTOME APOPTOSIS	no	CGP
PTEN	0.311020406	0.00075	0.00356	MG-132	Proteasome	START->CDKN2A(mutation)	REACTOME PI3K EVENTS IN ERBB4 SIGNALING,REACTOME PI3K EVENTS IN ERBB2 SIGNALING,BIOCARTA PTEN PATHWAY,REACTOME NEGATIVE REGULATION OF THE PI3K AKT NETWORK,REACTOME PI3K AKT ACTIVATION,KEGG P53 SIGNALING PATHWAY	no	CGP
PTEN	0.418588669	0.00415	0.01333	PLX4720	BRAF	START->BRAF(mutation)	REACTOME PI3K EVENTS IN ERBB4 SIGNALING,REACTOME PI3K EVENTS IN ERBB2 SIGNALING,BIOCARTA PTEN PATHWAY,REACTOME NEGATIVE REGULATION OF THE PI3K AKT NETWORK,REACTOME PI3K AKT ACTIVATION,KEGG P53 SIGNALING PATHWAY	no	CGP
PTK2	0.822207987	0.00425	0.01355	ABT-263	BCL2, BCL-XL, BCL-W	START->NOTCH1(mutation)	REACTOME APOPTOSIS,BIOCARTA PTEN PATHWAY	yes	CGP
PTK2	-0.30513158	0.0084	0.03128	AZD6244	MEK	START->BRAF(mutation)	REACTOME APOPTOSIS,BIOCARTA PTEN PATHWAY	yes	CCLE
PTK2	-0.39192545	0.0023	0.01075	PLX4720	RAF	START->BRAF(mutation)	REACTOME APOPTOSIS,BIOCARTA PTEN PATHWAY	yes	CCLE
PTK2	-0.3923164	0.0035	0.01546	RAF265	Raf kinase B, KDR	START->BRAF(mutation)	REACTOME APOPTOSIS,BIOCARTA PTEN PATHWAY	yes	CCLE
PTK2	-0.6095329	0.0015	0.00734	TAE684	ALK	START->HDHD1(deletion)	REACTOME APOPTOSIS,BIOCARTA PTEN PATHWAY	yes	CCLE
PTPN6	0.646745509	0.00265	0.00955	GDC-0449	SMO	START->EWS_FLI1(mutation)	KEGG JAK STAT SIGNALING PATHWAY	no	CGP
PTPN7	0.401730276	0.0045	0.0141	PLX4720	BRAF	START->BRAF(mutation)	KEGG MAPK SIGNALING PATHWAY	no	CGP
PTPRR	0.425960556	0.00085	0.00395	AKT_inhibitor_VIII	AKT1/2	START->PIK3CA(mutation)	KEGG MAPK SIGNALING PATHWAY	no	CGP
PTPRR	0.534601834	0.00495	0.01512	AZ628	BRAF	START->BRAF(mutation)	KEGG MAPK SIGNALING PATHWAY	no	CGP
RAC2	0.325949136	0.0004	0.0021	MG-132	Proteasome	START->CDKN2A(mutation)	KEGG WNT SIGNALING PATHWAY,KEGG MAPK SIGNALING PATHWAY	no	CGP
RASGRP1	0.306607289	0	0	RDEA119	MEK1/2	START->CDKN2A(mutation)	KEGG MAPK SIGNALING PATHWAY	no	CGP
RASGRP2	0.357754632	0.0002	0.00114	MG-132	Proteasome	START->CDKN2A(mutation)	KEGG MAPK SIGNALING PATHWAY	no	CGP
RPRM	0.515360332	0.0073	0.0199	AZ628	BRAF	START->BRAF(mutation)	KEGG P53 SIGNALING PATHWAY	no	CGP
RPS6KA2	0.301895892	0.00115	0.00503	PF-02341066	MET, ALK	START->CDKN2a(p14)(deletion)	KEGG MAPK SIGNALING PATHWAY,BIOCARTA MAPK PATHWAY	no	CGP
RPS6KA6	-0.30666078	0.00415	0.01785	AZD6244	MEK	START->BRAF(mutation)	KEGG MAPK SIGNALING PATHWAY	no	CCLE
RPS6KA6	-0.35264097	0.0012	0.006	PD-0325901	MEK	START->BRAF(mutation)	KEGG MAPK SIGNALING PATHWAY	no	CCLE
RPS6KA6	-0.33450132	0.00055	0.00293	PLX4720	RAF	START->BRAF(mutation)	KEGG MAPK SIGNALING PATHWAY	no	CCLE
RPS6KA6	-0.67281714	0.00795	0.03007	Topotecan	Topoisomerase I	START->IGLL5(deletion)	KEGG MAPK SIGNALING PATHWAY	no	CCLE

RRAS	-0.32777222	0.003	0.01355	AZD6244	MEK	START->BRAF(mutation)	KEGG MAPK SIGNALING PATHWAY	no	CCLE
RRAS	-0.325521	0.003	0.01355	PD-0325901	MEK	START->BRAF(mutation)	KEGG MAPK SIGNALING PATHWAY	no	CCLE
RRAS	0.406646046	0.0032	0.01105	SB590885	BRAF	START->BRAF(mutation)	KEGG MAPK SIGNALING PATHWAY	no	CGP
RRAS	0.312162675	0.00115	0.00503	Sunitinib	PDGFRA, PDGFRB, VEGFR(KDR), KIT, FLT3	START->CDKN2a(p14)(deletion)	KEGG MAPK SIGNALING PATHWAY	no	CGP
RRAS	-0.73559357	0	0	TAE684	ALK	START->HDHD1(deletion)	KEGG MAPK SIGNALING PATHWAY	no	CCLE
RRAS2	0.349566807	0.00885	0.02272	AKT_inhibitor_VIII	AKT1/2	START->PIK3CA(mutation)	KEGG MAPK SIGNALING PATHWAY	no	CGP
RRAS2	-0.45330648	0.00005	0.00029	AZD6244	MEK	START->BRAF(mutation)	KEGG MAPK SIGNALING PATHWAY	no	CCLE
RRAS2	-0.69020216	0.00135	0.00667	Lapatinib	EGFR, HER2	START->IKZF3(amplication)	KEGG MAPK SIGNALING PATHWAY	no	CCLE
RRAS2	0.405047387	0.0025	0.00916	MK-2206	AKT1/2	START->PIK3CA(mutation)	KEGG MAPK SIGNALING PATHWAY	no	CGP
RRAS2	-0.42431037	0.0001	0.00058	PD-0325901	MEK	START->BRAF(mutation)	KEGG MAPK SIGNALING PATHWAY	no	CCLE
RRAS2	-0.41768505	0.00115	0.00578	PLX4720	RAF	START->BRAF(mutation)	KEGG MAPK SIGNALING PATHWAY	no	CCLE
RRAS2	0.353903518	0.0087	0.02243	SB590885	BRAF	START->BRAF(mutation)	KEGG MAPK SIGNALING PATHWAY	no	CGP
RRAS2	0.339003585	0.0011	0.00486	Sunitinib	PDGFRA, PDGFRB, VEGFR(KDR), KIT, FLT3	START->CDKN2a(p14)(deletion)	KEGG MAPK SIGNALING PATHWAY	no	CGP
RRAS2	-0.82370913	0	0	TAE684	ALK	START->HDHD1(deletion)	KEGG MAPK SIGNALING PATHWAY	no	CCLE
SATB1	0.342200383	0.00025	0.0014	AZD-0530	SRC, ABL1	START->CDKN2A(mutation)	REACTOME APOPTOSIS	no	CGP
SATB1	0.300513231	0.00135	0.0057	BMS-536924	IGF1R	START->CDKN2A(mutation)	REACTOME APOPTOSIS	no	CGP
SATB1	0.316302055	0.00125	0.00537	Erlotinib	EGFR	START->CDKN2a(p14)(mutation)	REACTOME APOPTOSIS	no	CGP
SATB1	0.31001061	0.0005	0.00255	Erlotinib	EGFR	START->CDKN2A(mutation)	REACTOME APOPTOSIS	no	CGP
SERPINB5	0.823311889	0.0073	0.0199	ABT-263	BCL2, BCL-XL, BCL-W	START->NOTCH1(mutation)	KEGG P53 SIGNALING PATHWAY	yes	CGP
SERPINE1	0.508915291	0.00005	0.00032	AKT_inhibitor_VIII	AKT1/2	START->PIK3CA(mutation)	REACTOME SIGNALING BY TGF BETA RECEPTOR COMPLEX,KEGG P53 SIGNALING PATHWAY	yes	CGP
SFN	0.393781028	0.00615	0.01766	PLX4720	BRAF	START->BRAF(mutation)	KEGG CELL CYCLE,KEGG P53 SIGNALING PATHWAY	no	CGP
SFN	-0.43734983	0	0	PLX4720	RAF	START->BRAF(mutation)	KEGG CELL CYCLE,KEGG P53 SIGNALING PATHWAY	no	CCLE
SFN	0.386522537	0.00415	0.01333	SB590885	BRAF	START->BRAF(mutation)	KEGG CELL CYCLE,KEGG P53 SIGNALING PATHWAY	no	CGP
SFRP1	0.403398266	0.0023	0.0086	AKT_inhibitor_VIII	AKT1/2	START->PIK3CA(mutation)	KEGG WNT SIGNALING PATHWAY	no	CGP
SFRP1	-0.47663904	0.001	0.00508	Lapatinib	EGFR, HER2	START->ERBB2(amplication)	KEGG WNT SIGNALING PATHWAY	no	CCLE
SFRP1	-0.84909545	0.00135	0.00667	Topotecan	Topoisomerase I	START->NCRNA00226(deletion)	KEGG WNT SIGNALING PATHWAY	no	CCLE
SFRP4	-0.86398901	0.00865	0.03195	Irinotecan	Topoisomerase I	START->IGLL5(deletion)	KEGG WNT SIGNALING PATHWAY	no	CCLE
SMAD1	0.655329037	0.0093	0.02348	ABT-263	BCL2, BCL-XL, BCL-W	START->NOTCH1(mutation)	KEGG TGF BETA SIGNALING PATHWAY	no	CGP
SMAD1	-0.3123414	0.0057	0.02314	AZD6244	MEK	START->BRAF(mutation)	KEGG TGF BETA SIGNALING PATHWAY	no	CCLE
SMAD1	-0.30214294	0.00625	0.02492	PD-0325901	MEK	START->BRAF(mutation)	KEGG TGF BETA SIGNALING PATHWAY	no	CCLE
SMAD1	-0.41182663	0.00055	0.00293	PLX4720	RAF	START->BRAF(mutation)	KEGG TGF BETA SIGNALING PATHWAY	no	CCLE
SMAD1	-0.33423267	0.0051	0.02114	RAF265	Raf kinase B, KDR	START->BRAF(mutation)	KEGG TGF BETA SIGNALING PATHWAY	no	CCLE

SMAD7	0.397282639	0.0027	0.0097	AKT_inhibitor _VIII	AKT1/2	START->PIK3CA(mutation)	BIOCARTA TGFB PATHWAY,KEGG TGF BETA SIGNALING PATHWAY,PID TGFBRPATHWAY,REACTOME DOWNREGULATION OF TGF BETA RECEPTOR SIGNALING,REACTOME TGF BETA RECEPTOR SIGNALING ACTIVATES SMADS,REACTOME SIGNALING BY TGF BETA RECEPTOR COMPLEX	no	CGP
SMAD9	-0.79253586	0.0055	0.02248	Topotecan	Topoisomerase I	START->IGLL5(deletion)	KEGG TGF BETA SIGNALING PATHWAY	no	CCLE
SOCS1	-0.30211656	0.00415	0.01785	AZD6244	MEK	START->BRAF(mutation)	KEGG JAK STAT SIGNALING PATHWAY	no	CCLE
SOCS1	-0.34166386	0.00125	0.00623	PD-0325901	MEK	START->BRAF(mutation)	KEGG JAK STAT SIGNALING PATHWAY	no	CCLE
SOCS2	0.487417524	0.00835	0.02179	AZ628	BRAF	START->BRAF(mutation)	KEGG JAK STAT SIGNALING PATHWAY	no	CGP
SOX17	-0.33050948	0.0013	0.00645	AZD6244	MEK	START->BRAF(mutation)	KEGG WNT SIGNALING PATHWAY	no	CCLE
SOX17	-0.33249121	0.00175	0.00843	PD-0325901	MEK	START->BRAF(mutation)	KEGG WNT SIGNALING PATHWAY	no	CCLE
SOX17	0.762218707	0.00545	0.0162	ZM-447439	AURKB	START->NOTCH1(mutation)	KEGG WNT SIGNALING PATHWAY	no	CGP
SPRED1	-0.32011568	0.0072	0.02789	Panobinostat	HDAC	START->BRD2(mutation)	KEGG JAK STAT SIGNALING PATHWAY	no	CCLE
SPRY1	0.931688546	0.002	0.00772	Lapatinib	EGFR, ERBB2	START->SMAD4(mutation)	KEGG JAK STAT SIGNALING PATHWAY	no	CGP
SPRY2	0.755872577	0.0045	0.0141	ABT-263	BCL2, BCL-XL, BCL-W	START->NOTCH1(mutation)	KEGG JAK STAT SIGNALING PATHWAY	no	CGP
SPRY2	0.438737105	0.00105	0.00468	AKT_inhibitor _VIII	AKT1/2	START->PIK3CA(mutation)	KEGG JAK STAT SIGNALING PATHWAY	no	CGP
SPRY2	0.603041602	0.00745	0.02016	AZD-2281	PARP1/2	START->EWS_FLI1(mutation)	KEGG JAK STAT SIGNALING PATHWAY	no	CGP
SPRY4	0.351885149	0.00765	0.02053	AKT_inhibitor _VIII	AKT1/2	START->PIK3CA(mutation)	KEGG JAK STAT SIGNALING PATHWAY	no	CGP
SPRY4	-0.41696317	0.0044	0.01874	Panobinostat	HDAC	START->MAP4K4(mutation)	KEGG JAK STAT SIGNALING PATHWAY	no	CCLE
SPRY4	-0.30249674	0.00845	0.03141	Panobinostat	HDAC	START->BRD2(mutation)	KEGG JAK STAT SIGNALING PATHWAY	no	CCLE
ST3GAL6	0.481488653	0.00025	0.0014	AKT_inhibitor _VIII	AKT1/2	START->PIK3CA(mutation)	REACTOME SIGNALING BY NOTCH	no	CGP
ST3GAL6	-0.60896374	0.00155	0.00756	Lapatinib	EGFR, HER2	START->ERBB2(amplication)	REACTOME SIGNALING BY NOTCH	no	CCLE
ST3GAL6	-0.60230807	0.0003	0.00166	Lapatinib	EGFR, HER2	START->ZPBP2(amplication)	REACTOME SIGNALING BY NOTCH	no	CCLE
ST3GAL6	-0.59006932	0.0039	0.01696	Lapatinib	EGFR, HER2	START->IKZF3(amplication)	REACTOME SIGNALING BY NOTCH	no	CCLE
STAT4	0.421994595	0.00165	0.00662	AKT_inhibitor _VIII	AKT1/2	START->PIK3CA(mutation)	KEGG JAK STAT SIGNALING PATHWAY	no	CGP
STEAP3	0.384841932	0.00705	0.01943	AZD6244	MEK1/2	START->BRAF(mutation)	KEGG P53 SIGNALING PATHWAY	no	CGP
STEAP3	0.437684032	0.0059	0.01714	CI-1040	MEK1/2	START->BRAF(mutation)	KEGG P53 SIGNALING PATHWAY	no	CGP
STEAP3	0.889796646	0.00775	0.02071	Lapatinib	EGFR, ERBB2	START->SMAD4(mutation)	KEGG P53 SIGNALING PATHWAY	no	CGP
TBL1X	0.410952364	0.00335	0.01144	AZD6244	MEK1/2	START->BRAF(mutation)	KEGG WNT SIGNALING PATHWAY,REACTOME SIGNALING BY NOTCH	no	CGP
TBL1X	0.480186577	0.001	0.0045	PLX4720	BRAF	START->BRAF(mutation)	KEGG WNT SIGNALING PATHWAY,REACTOME SIGNALING BY NOTCH	no	CGP
TBL1X	-0.33811378	0.0033	0.0147	PLX4720	RAF	START->BRAF(mutation)	KEGG WNT SIGNALING PATHWAY,REACTOME SIGNALING BY NOTCH	no	CCLE
TBL1X	0.43285209	0.00115	0.00503	RDEA119	MEK1/2	START->BRAF(mutation)	KEGG WNT SIGNALING PATHWAY,REACTOME SIGNALING BY NOTCH	no	CGP
TBL1X	0.486496954	0.0004	0.0021	SB590885	BRAF	START->BRAF(mutation)	KEGG WNT SIGNALING PATHWAY,REACTOME SIGNALING BY NOTCH	no	CGP
TCF7L1	-0.48314226	0.00115	0.00578	Panobinostat	HDAC	START->MAP4K4(mutation)	KEGG WNT SIGNALING PATHWAY	no	CCLE
TCF7L1	-0.4368666	0.0051	0.02114	TAE684	ALK	START->HDHD1(deletion)	KEGG WNT SIGNALING PATHWAY	no	CCLE
TCF7L2	0.392543938	0.0045	0.0141	MK-2206	AKT1/2	START->PIK3CA(mutation)	KEGG WNT SIGNALING PATHWAY	no	CGP
TCF7L2	-0.48246792	0.00925	0.03356	TAE684	ALK	START->HDHD1(deletion)	KEGG WNT SIGNALING PATHWAY	no	CCLE

TGFB2	0.668156057	0.0048	0.01479	AZD-2281	PARP1/2	START->EWS_FLI1(mutation)	BIOCARTA TGFB PATHWAY,KEGG TGF BETA SIGNALING PATHWAY,PID TGFBRPATHWAY,KEGG MAPK SIGNALING PATHWAY,BIOCARTA MAPK PATHWAY,KEGG CELL CYCLE	no	CGP
TGFB2	-0.32357706	0.00345	0.01527	PD-0325901	MEK	START->BRAF(mutation)	BIOCARTA TGFB PATHWAY,KEGG TGF BETA SIGNALING PATHWAY,PID TGFBRPATHWAY,KEGG MAPK SIGNALING PATHWAY,BIOCARTA MAPK PATHWAY,KEGG CELL CYCLE	no	CCLE
TGFBR2	0.448672108	0.0006	0.00297	AKT_inhibitor_VIII	AKT1/2	START->PIK3CA(mutation)	BIOCARTA TGFB PATHWAY,KEGG TGF BETA SIGNALING PATHWAY,PID TGFBRPATHWAY,REACTOME DOWNREGULATION OF TGF BETA RECEPTOR SIGNALING,REACTOME TGF BETA RECEPTOR SIGNALING IN EMT EPITHELIAL TO MESENCHYMAL TRANSITION,REACTOME TGF BETA RECEPTOR SIGNALING ACTIVATES SMADS,REACTOME SIGNALING BY TGF BETA RECEPTOR COMPLEX,KEGG MAPK SIGNALING PATHWAY	yes	CGP
TGFBR2	0.883378516	0.00995	0.02461	Lapatinib	EGFR, ERBB2	START->SMAD4(mutation)	BIOCARTA TGFB PATHWAY,KEGG TGF BETA SIGNALING PATHWAY,PID TGFBRPATHWAY,REACTOME DOWNREGULATION OF TGF BETA RECEPTOR SIGNALING,REACTOME TGF BETA RECEPTOR SIGNALING IN EMT EPITHELIAL TO MESENCHYMAL TRANSITION,REACTOME TGF BETA RECEPTOR SIGNALING ACTIVATES SMADS,REACTOME SIGNALING BY TGF BETA RECEPTOR COMPLEX,KEGG MAPK SIGNALING PATHWAY	yes	CGP
TGFBR2	0.379961163	0.00555	0.01641	MK-2206	AKT1/2	START->PIK3CA(mutation)	BIOCARTA TGFB PATHWAY,KEGG TGF BETA SIGNALING PATHWAY,PID TGFBRPATHWAY,REACTOME DOWNREGULATION OF TGF BETA RECEPTOR SIGNALING,REACTOME TGF BETA RECEPTOR SIGNALING IN EMT EPITHELIAL TO MESENCHYMAL TRANSITION,REACTOME TGF BETA RECEPTOR SIGNALING ACTIVATES SMADS,REACTOME SIGNALING BY TGF BETA RECEPTOR COMPLEX,KEGG MAPK SIGNALING PATHWAY	yes	CGP
TGFBR3	0.346147062	0.00905	0.02306	AKT_inhibitor_VIII	AKT1/2	START->PIK3CA(mutation)	PID TGFBRPATHWAY	no	CGP
TGIF1	0.87433197	0.00045	0.00233	ABT-263	BCL2, BCL-XL, BCL-W	START->NOTCH1(mutation)	REACTOME SIGNALING BY TGF BETA RECEPTOR COMPLEX	no	CGP
TGIF1	0.669562495	0.00315	0.01091	AZD-2281	PARP1/2	START->EWS_FLI1(mutation)	REACTOME SIGNALING BY TGF BETA RECEPTOR COMPLEX	no	CGP
TGIF1	0.646963134	0.0039	0.01278	GDC-0449	SMO	START->EWS_FLI1(mutation)	REACTOME SIGNALING BY TGF BETA RECEPTOR COMPLEX	no	CGP
TGIF1	0.470795681	0.0007	0.00338	MK-2206	AKT1/2	START->PIK3CA(mutation)	REACTOME SIGNALING BY TGF BETA RECEPTOR COMPLEX	no	CGP
TGIF1	0.626858696	0.00555	0.01641	Vorinostat	HDAC inhibitor Class I, IIa, IIb, IV	START->EWS_FLI1(mutation)	REACTOME SIGNALING BY TGF BETA RECEPTOR COMPLEX	no	CGP
THBS2	0.37281938	0.00405	0.0131	AKT_inhibitor_VIII	AKT1/2	START->PIK3CA(mutation)	KEGG TGF BETA SIGNALING PATHWAY	no	CGP
THBS4	-0.60052019	0.00855	0.03167	Lapatinib	EGFR, HER2	START->ZPBP2(amplication)	KEGG TGF BETA SIGNALING PATHWAY	no	CCLE
THBS4	0.646046702	0.0009	0.00413	ZM-447439	AURKB	START->NOTCH1(mutation)	KEGG TGF BETA SIGNALING PATHWAY	no	CGP
TIMP3	-0.59428373	0.00955	0.03437	Lapatinib	EGFR, HER2	START->IKZF3(amplication)	BIOCARTA P53 PATHWAY	no	CCLE
TIMP3	-0.35512354	0.0029	0.01316	Panobinostat	HDAC	START->BRD2(mutation)	BIOCARTA P53 PATHWAY	no	CCLE
TJP1	0.362108941	0.0009	0.00413	PF-02341066	MET, ALK	START->CDKN2a(p14)(deletion)	REACTOME APOPTOSIS	no	CGP
TJP1	-0.35271708	0.0035	0.01546	PLX4720	RAF	START->BRAF(mutation)	REACTOME APOPTOSIS	no	CCLE
TJP1	0.30497979	0.004	0.013	Sunitinib	PDGFRA, PDGFRB, VEGFR(KDR), KIT, FLT3	START->CDKN2a(p14)(deletion)	REACTOME APOPTOSIS	no	CGP
TJP1	-0.55365523	0.0038	0.01659	TAE684	ALK	START->HDHD1(deletion)	REACTOME APOPTOSIS	no	CCLE
TJP2	0.646492274	0.0002	0.00114	AZ628	BRAF	START->BRAF(mutation)	REACTOME APOPTOSIS	no	CGP
TJP2	0.369423916	0.0001	0.00061	AZ628	BRAF	START->CDKN2A(mutation)	REACTOME APOPTOSIS	no	CGP
TJP2	0.414205496	0.0047	0.01457	PLX4720	BRAF	START->BRAF(mutation)	REACTOME APOPTOSIS	no	CGP

TJP2	0.501038646	0.0001	0.00061	SB590885	BRAF	START->BRAF(mutation)	REACTOME APOPTOSIS	no	CGP
TJP2	0.721555236	0.00805	0.02127	ZM-447439	AURKB	START->NOTCH1(mutation)	REACTOME APOPTOSIS	no	CGP
TLE2	-0.45159588	0	0	PLX4720	RAF	START->BRAF(mutation)	REACTOME SIGNALING BY NOTCH	no	CCLE
TLE4	0.301897746	0.0011	0.00486	Erlotinib	EGFR	START->CDKN2A(mutation)	REACTOME SIGNALING BY NOTCH	no	CGP
TNFSF10	0.444338831	0.00015	0.00089	SB590885	BRAF	START->BRAF(mutation)	REACTOME APOPTOSIS,KEGG APOPTOSIS	no	CGP
TP53I3	-0.40967343	0.00585	0.02363	Panobinostat	HDAC	START->MAP4K4(mutation)	KEGG P53 SIGNALING PATHWAY	no	CCLE
UNC5B	-0.30338764	0.00565	0.02297	PD-0325901	MEK	START->BRAF(mutation)	REACTOME REGULATION OF APOPTOSIS,REACTOME APOPTOSIS	no	CCLE
UNC5B	-0.38008983	0.00015	0.00085	PLX4720	RAF	START->BRAF(mutation)	REACTOME REGULATION OF APOPTOSIS,REACTOME APOPTOSIS	no	CCLE
WNT5B	0.616758761	0.00665	0.01865	ZM-447439	AURKB	START->NOTCH1(mutation)	KEGG WNT SIGNALING PATHWAY,KEGG HEDGEHOG SIGNALING PATHWAY	no	CGP
WNT7A	-0.60934552	0.00165	0.008	Lapatinib	EGFR, HER2	START->ZPBP2(amplication)	KEGG WNT SIGNALING PATHWAY,KEGG HEDGEHOG SIGNALING PATHWAY,PID WNT SIGNALING PATHWAY	no	CCLE
WWTR1	0.332267977	0	0	PD-0332991	CDK4/6	START->CDKN2a(p14)(mutation)	REACTOME SIGNALING BY TGF BETA RECEPTOR COMPLEX	no	CGP
WWTR1	0.322153085	0.00095	0.00432	Sunitinib	PDGFRA, PDGFRB, VEGFR(KDR), KIT, FLT3	START->CDKN2a(p14)(deletion)	REACTOME SIGNALING BY TGF BETA RECEPTOR COMPLEX	no	CGP
WWTR1	-0.62747544	0.00015	0.00085	TAE684	ALK	START->HDHD1(deletion)	REACTOME SIGNALING BY TGF BETA RECEPTOR COMPLEX	no	CCLE
YAP1	-0.34911214	0.00435	0.01857	PLX4720	RAF	START->BRAF(mutation)	PID TGFBRPATHWAY	no	CCLE
YAP1	-0.38476715	0.00235	0.01096	RAF265	Raf kinase B, KDR	START->BRAF(mutation)	PID TGFBRPATHWAY	no	CCLE
YAP1	-0.54628922	0.0069	0.02699	TAE684	ALK	START->HDHD1(deletion)	PID TGFBRPATHWAY	no	CCLE
YES1	0.828247767	0.0037	0.01229	ABT-263	BCL2, BCL-XL, BCL-W	START->NOTCH1(mutation)	PID WNT NONCANONICAL PATHWAY	yes	CGP
ZIC2	-0.75716928	0.00215	0.01012	Lapatinib	EGFR, HER2	START->ZPBP2(amplication)	KEGG HEDGEHOG SIGNALING PATHWAY	no	CCLE
ZMAT3	0.592777574	0.00005	0.00032	AKT_inhibitor_VIII	AKT1/2	START->PIK3CA(mutation)	KEGG P53 SIGNALING PATHWAY	no	CGP
